# Broad-scale sampling of primary freshwater fish populations reveals the role of intrinsic traits, inter-basin connectivity, drainage area and latitude on shaping contemporary patterns of genetic diversity

**DOI:** 10.7717/peerj.1694

**Published:** 2016-02-29

**Authors:** Carla Sousa-Santos, Joana I. Robalo, Ana M. Pereira, Paulo Branco, José Maria Santos, Maria Teresa Ferreira, Mónica Sousa, Ignacio Doadrio

**Affiliations:** 1 MARE, ISPA, Lisbon, Portugal; 2 CEF—Centro de Estudos Florestais, Instituto Superior de Agronomia, Universidade de Lisboa, Lisbon, Portugal; 3 CERis—Civil Engineering Research and Innovation for Sustainability, Instituto Superior Técnico, Lisbon, Portugal; 4 Instituto da Conservação da Natureza e das Florestas, I.P., Lisbon, Portugal; 5 Museo Nacional de Ciencias Naturales, CSIC, Madrid, Spain

**Keywords:** Cyprinidae, Haplotype diversity, Nucleotide diversity, Mediterranean streams, Freshwater fish conservation, Genetic diversity drivers, Endangered species

## Abstract

**Background.** Worldwide predictions suggest that up to 75% of the freshwater fish species occurring in rivers with reduced discharge could be extinct by 2070 due to the combined effect of climate change and water abstraction. The Mediterranean region is considered to be a hotspot of freshwater fish diversity but also one of the regions where the effects of climate change will be more severe. Iberian cyprinids are currently highly endangered, with over 68% of the species raising some level of conservation concern.

**Methods.** During the FISHATLAS project, the Portuguese hydrographical network was extensively covered (all the 34 river basins and 47 sub-basins) in order to contribute with valuable data on the genetic diversity distribution patterns of native cyprinid species. A total of 188 populations belonging to 16 cyprinid species of *Squalius, Luciobarbus, Achondrostoma, Iberochondrostoma, Anaecypris* and *Pseudochondrostoma* were characterized, for a total of 3,678 cytochrome *b* gene sequences.

**Results.** When the genetic diversity of these populations was mapped, it highlighted differences among populations from the same species and between species with identical distribution areas. Factors shaping the contemporary patterns of genetic diversity were explored and the results revealed the role of latitude, inter-basin connectivity, migratory behaviour, species maximum size, species range and other species intrinsic traits in determining the genetic diversity of sampled populations. Contrastingly, drainage area and hydrological regime (permanent vs. temporary) seem to have no significant effect on genetic diversity. Species intrinsic traits, maximum size attained, inter-basin connectivity and latitude explained over 30% of the haplotype diversity variance and, generally, the levels of diversity were significantly higher for smaller sized species, from connected and southerly river basins.

**Discussion.** Targeting multiple co-distributed species of primary freshwater fish allowed us to assess the relative role of historical *versus* contemporary factors affecting genetic diversity. Since different patterns were detected for species with identical distribution areas we postulate that contemporary determinants of genetic diversity (species’ intrinsic traits and landscape features) must have played a more significant role than historical factors. Implications for conservation in a context of climate change and highly disturbed habitats are detailed, namely the need to focus management and conservation actions on intraspecific genetic data and to frequently conduct combined genetic and demographic surveys.

## Introduction

Freshwater biodiversity has declined faster than terrestrial and marine biodiversity over the last decades (Jenkins, 2003). Up to 75% of the freshwater fish species occurring in rivers with reduced flow could be extinct by 2070 due to climate change and water abstraction (Xenopoulos et al., 2005). The Iberian freshwater ichthyofauna is very rich in diversity and endemisms (Doadrio et al., 2011), a feature that was potentiated by geographical isolation and that is common to other Mediterranean peninsulas (Clavero, Blanco-Garrido & Prenda, 2004). Cyprinids are the most diverse and ecologically important components of the Iberian native ichthyofauna, contributing to the Mediterranean hotspot of freshwater fish diversity (Myers et al., 2000) with at least 16 endemic species (Doadrio et al., 2011). However, native cyprinids are highly endangered, with over 68% (26 out of 38) of the species raising some level of conservation concern (Cabral et al., 2005; Doadrio et al., 2011).

Most endangered Iberian cyprinids have extremely restricted geographical ranges and occur in temporary Mediterranean-type Rivers with autumn-winter floods and extended summer droughts, resulting in a series of disconnected pools (Gasith & Resh, 1999; Alvarez-Cobelas, Rojo & Angeler, 2005). Although these pools act as summer refugia, the congregation of fish in these pools also results in increased predation, high competition for limited space and food, low concentration of oxygen, high water temperature, and a higher probability of being affected by infectious diseases (Magoulick & Kobza, 2003; Dekar & Magoulick, 2007). Thus, summer droughts are often responsible for population fragmentation and depletion that cyclically affect the structure of these freshwater fish communities (Magalhães et al., 2002). Moreover, endemic cyprinids face the direct and indirect effects of continuous and multiple anthropogenic threats: pollution, damming, habitat loss or degradation and proliferation of exotic species. As a consequence, populations of cyprinids are suffering a generalised decline (Macedo-Veiga, 2013) that will likely increase with the effects of climate change, expected to be particularly severe in Mediterranean climate regions (Schröter et al., 2005). Indeed, both gradual climatic changes and extreme events are likely to impact freshwater fish populations, resulting in drastic reductions and/or changes in species-distribution ranges, communities and life-histories that ultimately may lead to extinction (Filipe, Lawrence & Bonada, 2013). Migrating or dispersing to more favourable sites might obviate extinction. However, as Iberian cyprinids are primary freshwater fish, they are confined to their habitats and, thus, their evolutionary history closely resembles the evolution of paleodrainages and the rearrangements of the fluvial network through time (Reyjol et al., 2007). This obligatory confinement makes them excellent models to study speciation and the radiation of ancient lineages throughout Iberia (e.g., Salgueiro et al., 2003; Mesquita et al., 2005; Sousa et al., 2007; Almada & Sousa-Santos, 2010; Gante, 2011; Lopez-Cunha et al., 2012; Aboim et al., 2013; Sousa-Santos et al., 2014a; Sousa-Santos et al., 2014b).

Although crucial, the application of genetics in the management of wild threatened species is still far from being common (Frankham, 2010). Thus, given the imperilment of most of the Portuguese native cyprinid species, the FISHATLAS project was launched to contribute with valuable data on the genetic diversity distribution patterns of native cyprinid species that would help to prioritize target populations for conservation.

As the distribution of the genetic diversity may have been shaped by historical and contemporary events we aimed to disentangle the factors underlying the observed patterns. As such, in parallel with the spatial patterning of genetic diversity, the broad-scale sampling also allowed for testing the effects of species intrinsic traits and environmental characteristics on observed levels of genetic diversity. Data obtained allowed us to address the following questions: (1) Is the genetic diversity of endemic fish populations influenced by the specific status of each population and by other characteristics that are intrinsic to the species, such as the maximum size or migratory behaviour? (2) Is the genetic diversity of each population influenced by the area and by the hydrological regime of the river drainages they inhabit? (3) Does latitude influence genetic diversity, given that populations from northern rivers are subjected to lower temperatures and fewer temperature fluctuations, contrasting with those from southern rivers which are exposed to higher temperatures, lower oxygen concentrations and cyclical regimes of floods and droughts (Magalhães, Schlosser & Collares-Pereira, 2003; Henriques, Sousa & Coelho, 2010; Füssel et al., 2012; Jesus, Inácio & Coelho, 2013)? (4) Do populations of species with wider distribution ranges show higher overall genetic diversity than those with more geographically confined distributions? and (5) do populations inhabiting isolated drainages show less genetic diversity than those occupying interconnected sub-basins of a dendritic river basin?

## Materials and Methods

### Target species

A total of 17 Iberian endemic cyprinids were selected as target species: *Anaecypris hispanica* (Steindachner, 1866), *Achondrostoma oligolepis* (Robalo, Doadrio, Almada & Kottelat, 2005), *Achondrostoma occidentale* (Robalo, Almada, Sousa-Santos, Moreira & Doadrio, 2005), *Iberochondrostoma lemmingii* (Steindachner, 1866), *Iberochondrostoma lusitanicum* (Collares-Pereira, 1980), *Iberochondrostoma almacai* (Coelho, Mesquita & Collares-Pereira, 2005), *Pseudochondrostoma polylepis* (Steindachner, 1865), *Pseudochondrostoma duriense* (Coelho, 1985), *Pseudochondrostoma willkommii* (Steindachner, 1866), *Luciobarbus microcephalus* (Almaça, 1967), *Luciobarbus bocagei* (Steindachner, 1865), *Luciobarbus sclateri* (Günther, 1868), *Luciobarbus comizo* (Steindachner, 1865), *Squalius carolitertii* (Doadrio, 1987), *Squalius pyrenaicus* (Günther, 1868), *Squalius torgalensis* (Bogutskaya, Rodrigues & Collares-Pereira, 1998) and *Squalius aradensis* (Bogutskaya, Rodrigues & Collares-Pereira, 1998). Of all the cyprinids native to Portugal, only four species [*Squalius alburnoides* (Steindachner, 1866)*, Luciobarbus steindachneri* (Almaça, 1967), *Achondrostoma arcasii* (Steindachner, 1866) and *Iberochondrostoma olisiponensis* (Gante, Santos & Alves, 2007)] were not included *a priori* due to their hybridogenetic origin (Sousa-Santos, Collares-Pereira & Almada, 2007), uncertain taxonomic classification (Robalo et al., 2006; Gante et al., 2015) or extreme scarcity in wild populations (Sousa-Santos et al., 2014a). *L. microcephalus* was posteriorly excluded from the analyses due to the low number of individuals sampled in each population (see below).

### Sampling

**Figure 1:**
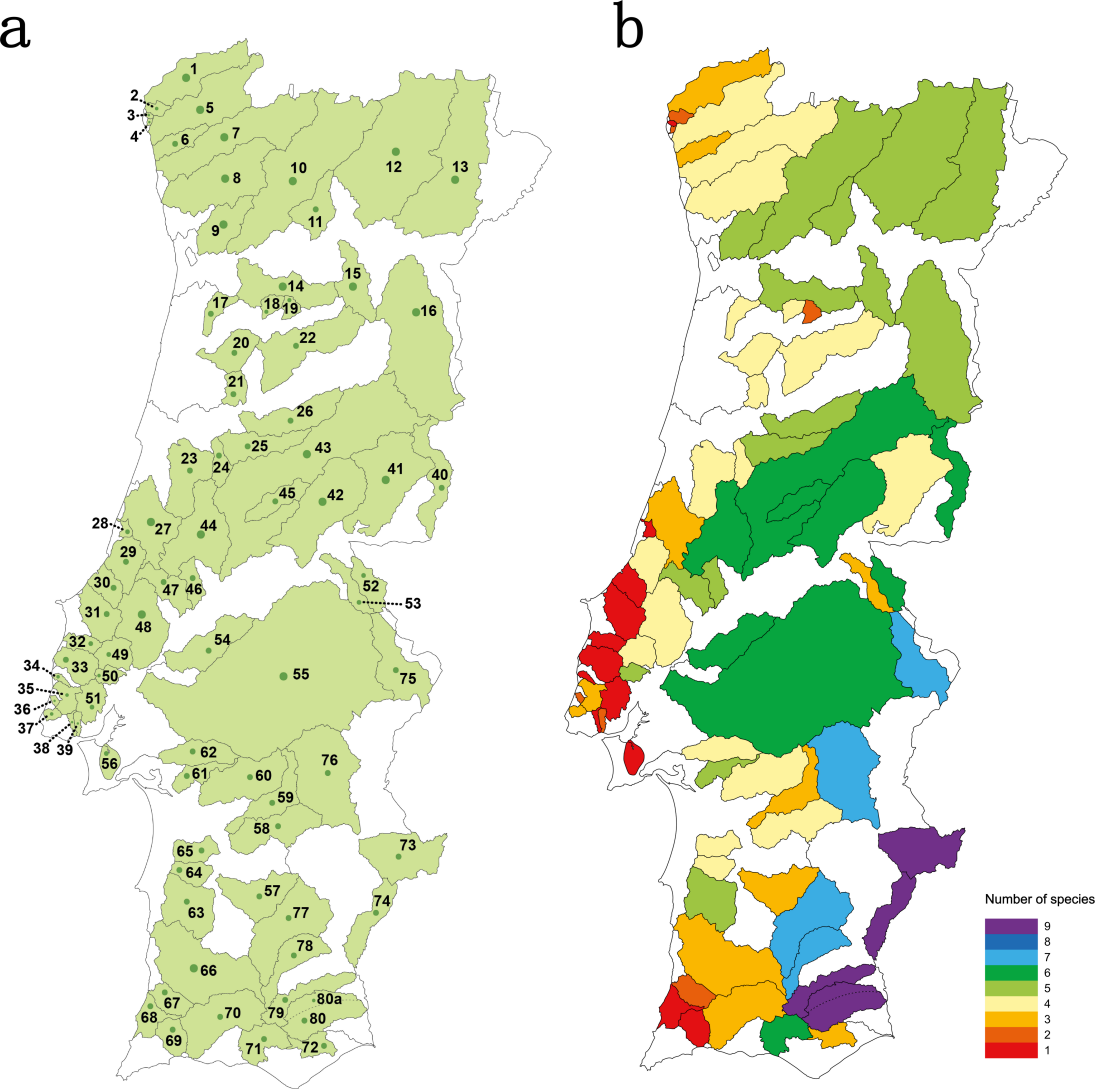
Studied area. (A) sampled river basins and sub-basins; (B) number of native cyprinid species occurring in each sampled river basin/sub-basin. Legend: 1-Minho, 2-Âncora, 3-Cabanas, 4-Pego, 5-Lima, 6-Neiva, 7-Cávado, 8-Ave, 9-Douro–Sousa, 10-Douro–Tâmega, 11-Douro–Corgo, 12-Douro–Tua, 13-Douro–Sabor, 14-Douro–Paiva, 15-Douro–Távora, 16-Douro–Coa, 17-Vouga–Caima, 18-Vouga–Sul, 19-Vouga–Mel, 20-Vouga–Águeda, 21- Mondego–Mortágua, 22-Mondego–Dão, 23-Mondego–Arunca, 24-Mondego–Corvo, 25-Mondego–Ceira, 26-Mondego–Alva, 27-Lis, 28-São Pedro, 29-Alcoa, 30-Tornada, 31-Real, 32-Alcabrichel, 33-Sizandro, 34-Safarujo, 35-Lizandro, 36-Samarra, 37-Colares, 38-Barcarena, 39-Jamor, 40-Tagus–Erges, 41-Tagus–Ponsul, 42-Tagus–Ocreza, 43-Tagus–Zezere, 44-Tagus–Zêzere Nabão, 45-Tagus–Zezere Sertã, 46-Tagus–Almonda, 47-Tagus–Alviela, 48-Tagus–Maior, 49-Tagus–Ota, 50-Tagus–Grande da pipa, 51-Tagus–Trancão, 52-Tagus–Sever, 53-Tagus–Nisa, 54-Tagus–Muge, 55-Tagus–Sorraia, 56-Tagus–Coina, 57-Sado–Roxo, 58-Sado–Odivelas, 59-Sado–Xarrama, 60-Sado–Alcaçovas, 61-Sado–S.Martinho, 62-Sado–Marateca, 63-Sado–Campilhas, 64-Sado–Corona, 65-Sado–Grândola, 66-Mira, 67-Seixe, 68-Aljezur, 69-Alvor, 70-Arade, 71-Quarteira, 72-Gilão, 73-Guadiana–Ardila, 74-Guadiana–Chança, 75-Guadiana–Caia, 76-Guadiana–Degebe, 77-Guadiana–Cobres, 78-Guadiana–Oeiras, 79-Guadiana–Vascão, 80-Guadiana–Odeleite, 80a-Guadiana–Foupana.

Thirty four river basins were sampled in the Portuguese mainland hydrographical network. The six largest river basins (Douro, Vouga, Mondego, Tagus, Sado and Guadiana) were further sub-divided into 47 sub-basins resulting in a total number of 81 geographical units sampled (Table S1 and Fig. 1A). Populations for which less than 15 individuals were collected were excluded from analyses.

Fish were collected with standard wadable electrofishing procedures (CEN, 2003) and returned to the water immediately after non-destructive sampling. Collected fin clips were preserved in 96% ethanol and vouchers were kept at the tissue collection of MARE/ISPA for subsequent DNA extraction, amplification and sequencing. Permits for field work were given by ICNF (permit number 176/2010/CAPT and 53/2012/CAPT).

### DNA extraction, amplification and sequencing

Total genomic DNA was extracted from fin clips using REDExtract-N-Amp Tissue PCR kits (Sigma-Aldrich) following the manufacturer’s instructions. The mitochondrial cytochrome *b* (cyt*b*) gene was amplified using the primers LCB1-new ACTTGAAGAACCACCGTTG (adapted from the LCB1 primer described by Brito et al., 1997) and HA-CAACGATCTCCGGTTTACAAGAC (Schmidt & Gold, 1993). PCR conditions were the following: 35 × (94°C1′ + 50°C1′ + 72°C2′). PCR products were purified and sequenced in the forward direction using the LCB1-new primer, at GATC Biotech (Konstanz, Germany). Obtained sequences were trimmed at the 3′ and 5′ ends so they had the same length for all the individuals sampled (720bp) and deposited in GenBank ( KU366823– KU370500).

### DNA analyses and gene diversity mapping

All sequences were edited and aligned using CodonCode Aligner v4.0.4 (CodonCode Corp., USA). The search for shared haplotypes and the listing of representative sequences was conducted by DNAcollapser (FaBox v.1.41; http://www.birc.au.dk/software/fabox).

ARLEQUIN software package V.3.5 (Excoffier & Lischer, 2010) was used to quantify the number of private haplotypes for each population (i.e., haplotypes that were exclusive for the considered population and that were not found elsewhere). These values were then used to calculate the percentage of private haplotypes *per* population (of all the haplotypes found in the considered population) and the average percentage of private haplotypes *per* population for each target species (%*N*_*PH*_). Mean, standard deviation, minimum and maximum values of %*N*_*PH*_ were obtained with EXCEL 2013 (Microsoft^®^).

ARLEQUIN was also used to estimate gene diversity (*h* index, defined as the probability that two randomly chosen haplotypes are different in a sample; used as a measure of haplotype diversity for haploid data), nucleotide diversity (*π* index, defined as the probability that two randomly chosen homologous nucleotides are different) and mean number of pairwise differences (MNPD, defined as the mean number of differences between all pairs of haplotypes in a sample) for each population. Analyses of molecular variance (AMOVA) were also performed with ARLEQUIN.

Values obtained for the *h* index were mapped in the sampling areas by Yris Graphics (www.yrisgraphics.com). This index, which varies between 0 and 1, reflects the probability of two randomly chosen haplotypes being different in a sample, and was selected to illustrate haplotype diversity of sampled populations. Using this index, diversity classes (common for all species) were established, allowing for an automatic visual inspection of the diversity level of distinct populations. The maps and database are available for download at the project’s webpage (www.fishatlas.net).

### Data analyses and hypothesis testing

Populations, regardless of species, were considered the unit of comparison. In order to test whether the obtained genetic diversity pattern was influenced by species’ intrinsic traits and extrinsic factors, data concerning 8 variables were collected and organized in a matrix (Table S2). Six of these variables are categorical: (1) “species” (with 16 categories, corresponding to the 16 species studied: *L. bocagei, L. sclateri, L. comizo, P. duriense, P. polylepis, P. willkommii, I. almacai, I. lusitanicum, I. lemmingii, S. carolitertii, S. pyrenaicus, S. aradensis, S. torgalensis, A. hispanica, A. oligolepis* and *A. occidentale*), and the dichotomous variables (2) “hydrological regime,” (3) “latitude,” (4) “migratory behaviour,” (5) “species range,” and (6) “inter-basin connectivity.” The quantitative variables “drainage area” and “species maximum size” were also included in the matrix.

“Hydrological regime” was classified as either permanent or temporary, depending on whether or not the population occurs in river basins that maintain flowing water throughout the year; “latitude” as northern or southern (for river basins located north or south of the Central Massif of Estrela, which divides the Portuguese territory in two halves of distinct reliefs and climate); “migratory behaviour” as non-migratory or potamodromous (using ecological data compiled by Ribeiro et al., 2007); “species range” as wide or restricted if the species occurs, respectively, in more or less than 12 river basins/sub-basins (see Sousa-Santos et al., 2013 for the distribution areas of each species); and “inter-basin connectivity” as connected or unconnected to other water bodies. Drainage areas were calculated from the shape file of Portuguese hydrographical network (downloaded from http://sniamb.apambiente.pt/Home/Default.htm) by using the polygon area as implemented in QGIS 2.10.1 software. Whenever a population inhabits a sub-basin of a larger basin, the area of the sub-drainage was considered, as for the studied small fish species the main course of a large river may represent a natural barrier to gene flow.

Individual linear regressions were performed to test the effect of each of these independent variables on *h*, *π* and MNPD indices calculated for each population. Dummy variables were created for the categorical independent variable “species,” enabling the use of the 16 categories of this variable in a regression model.

Predictor variables with significant effects (*α* < 0.05) were selected as candidate variables for the modelling process. Then, a hierarchical linear regression was performed, first including all selected variables (excluding “species”) with a stepwise method and, finally including the categorical variable “species.” This procedure sought to first analyse the effect of each of the variables regardless of the effect of “species.” This variable was then included to test whether other aspects intrinsic to the species, not measured by the remaining independent variables, were significant. This method was adopted independently for each of the three dependent variables: *h*, *π* and MNPD. We search for the presence of outliers and excluded them to avoid spurious trends or masking of valid ones.

For the analyses of each species separately, given the lower number of samples, non-parametric tests were performed: Spearman’s *ρ*, to test the correlation between “drainage area” and each of the dependent variables; and Mann–Whitney’s *U* tests to compare groups (populations inhabiting connected or unconnected water bodies; populations occurring in permanent or temporary river basins; and northern and southern populations) concerning their genetic diversity (*h*, *π* and MNPD indices). All statistical analyses were conducted using IBM SPSS Statistics, version 22 (IBM Corporation, 2013).

## Results

Species richness concerning the Portuguese native cyprinid ichthyofauna varied between 1 and 9 species *per* river basin/sub-basin (Fig. 1B). The larger river basins, such as those of the Douro, Tagus and Guadiana, accommodate the highest values of species richness, while in the smaller coastal river basins draining into the Atlantic only one to three native cyprinids were found (Fig. 1B).

### Patterns of genetic diversity

In general, populations of the small sized non-migratory species *A. hispanica*, *A. oligolepis, I. lemmingii, I. lusitanicum, I. almacai, S. aradensis, S. carolitertii* and *S. pyrenaicus* show higher percentages of private haplotypes (average values *per* species ranging from 27.84% to 75.00%, Table 1) than populations of the larger sized potamodromous species *L. bocagei, L. comizo, L. sclateri, P. willkommii, P. duriense* and *P. polylepis* (average values *per* species ranging from 2.14% to 25.00%, Table 1).

**Table 1:** Genetic diversity of populations. Number of sampled individuals (*N*_*IND*_), number of sampled populations (*N*_*POP*_), number of haplotypes retrieved (*N*_*H*_) and average percentage of private haplotypes per population (%*N*_*PH*_), for each target species. Values obtained for haplotype diversity (*h*), nucleotide diversity (*π*) and mean number of pairwise differences (MNPD) are also presented.

Species	*N*_*IND*_	*N*_*POP*_	*N*_*H*_	%*N*_*PH*_ [mean ± sd (min–max)]	*h*	*π*	MNPD
*A. hispanica*	109	5	36	46.76 ± 15.01% (22.22%–60.00%)	0.936 ± 0.010	0.005 ± 0.003	3.625 ± 1.852
*A. occidentale*	72	3	9	45.24 ± 43.06% (0%–85.71%)	0.752 ± 0.033	0.002 ± 0.002	1.655 ± 0.986
*A. oligolepis*	501	26	47	27.84 ± 31.18% (0%–87.50%)	0.824 ± 0.016	0.012 ± 0.007	9.301 ± 4.281
*I. lemmingii*	57	3	10	75.00 ± 0%	0.710 ± 0.057	0.002 ± 0.002	1.780 ± 1.045
*I. lusitanicum*	297	14	25	32.74 ± 33.73% (0%–100%)	0.827 ± 0.016	0.008 ± 0.004	5.835 ± 2.797
*I. almacai*	40	2	4	41.67 ± 11.79% (33.33%–50.00%)	0.512 ± 0.080	0.001 ± 0.001	0.573 ± 0.474
*S. carolitertii*	430	21	34	35.48 ± 32.73% (0%–100%)	0.704 ± 0.022	0.006 ± 0.003	4.524 ± 2.230
*S. aradensis*	98	5	11	29.33 ± 40.44% (0%–80.00%)	0.739 ± 0.025	0.004 ± 0.002	2.587 ± 1.398
*S. pyrenaicus*	343	18	83	48.81 ± 30.59% (0%–85.71%)	0.930 ± 0.007	0.014 ± 0.007	10.080 ± 4.616
*S. torgalensis*	21	1	4	–	0.271 ± 0.124	0.001 ± 0.001	1.038 ± 0.720
*L. bocagei*	716	39	11	2.14 ± 9.50% (0%–50.00%)	0.386 ± 0.022	0.001 ± 0.001	0.537 ± 0.447
*L. comizo*	130	6	4	25.00 ± 27.39% (0%–50.00%)	0.061 ± 0.029	0.000 ± 0.000	0.061 ± 0.133
*L. sclateri*	209	9	7	14.81 ± 22.74% (0%–50.00%)	0.311 ± 0.040	0.001 ± 0.001	0.815 ± 0.588
*P. willkommii*	113	6	16	17.02 ± 10.11% (0%–28.57%)	0.818 ± 0.024	0.002 ± 0.002	1.793 ± 1.044
*P. duriense*	254	14	22	15.82 ± 22.77% (0%–71.43%)	0.892 ± 0.007	0.003 ± 0.002	2.181 ± 1.212
*P. polylepis*	288	16	26	24.43 ± 19.97% (0%–50.00%)	0.747 ± 0.018	0.002 ± 0.001	1.407 ± 0.865
Total	3,678	188	349				

The average values of haplotype diversity ranged between *h* = 0.061 ± 0.029 (for *L. comizo*) and *h* = 0.936 ± 0.010 for (*A. hispanica*). The average nucleotide diversity and the average mean number of pairwise differences showed the same pattern (Table 1): *L. comizo* presented the lowest values (*π* = 0.000 ± 0.000 and MNPD = 0.061 ± 0.133, respectively) and *S. pyrenaicus* the highest (*π* = 0.014 ± 0.007 and MNPD = 10.080 ± 4.616, respectively).

**Figure 2:**
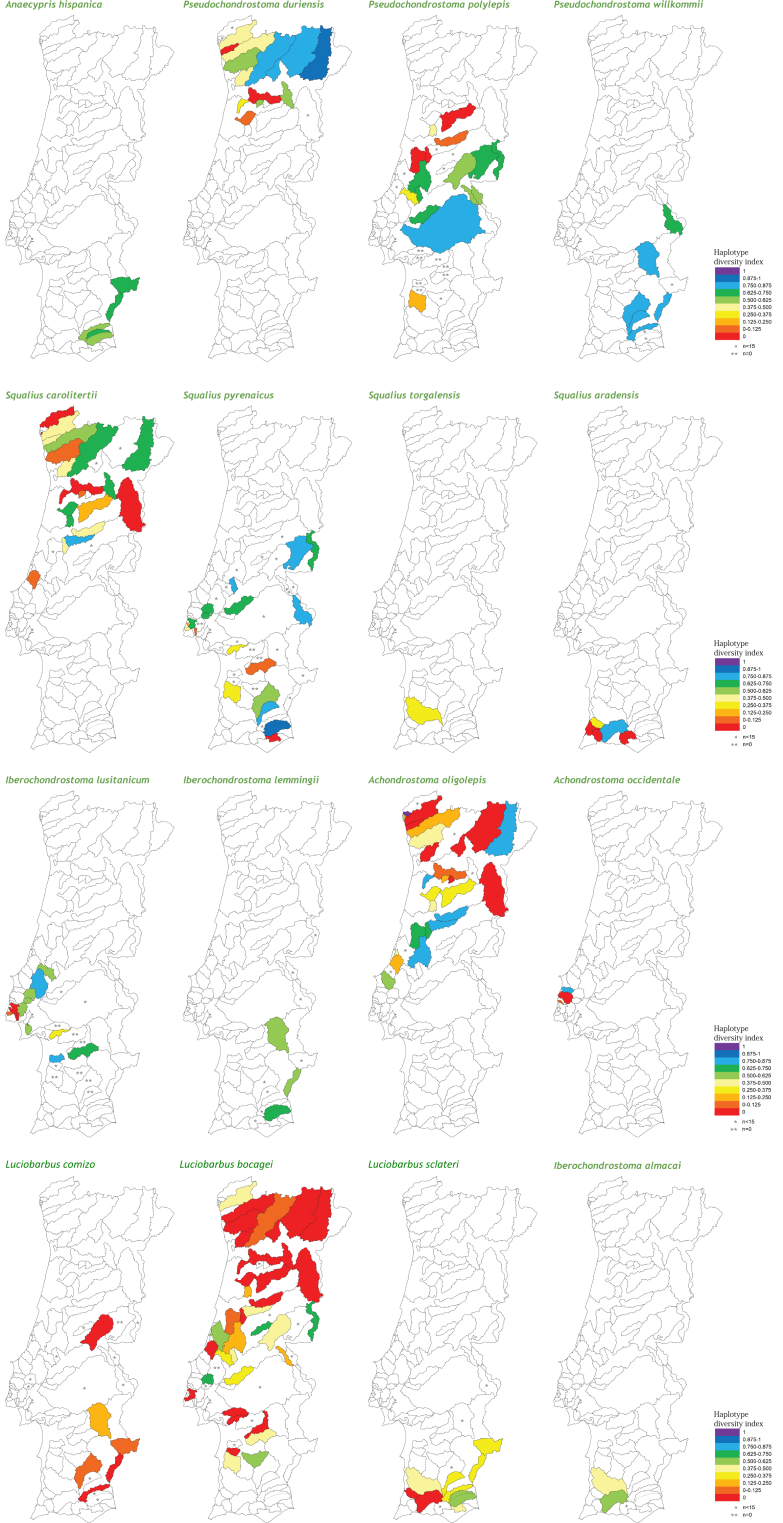
Genetic diversity mapping. Spatial distribution of the haplotype diversity (*h*) values obtained for each population of the target species.

At the population level, the spatial distribution of haplotype diversity values revealed distinct levels of diversity among populations of the same species and distinct patterns among species (Fig. 2). Indeed, analyses of molecular variance (AMOVAs) conducted for each target species separately revealed two contrasting patterns: in eight of the 15 species most of the variation (ranging from 50.65% for *L. bocagei* to 91.91% for *A. oligolepis*) could be attributed to differences among populations, while for the remaining seven species 53.26% (for *P. polylepis*) to 98.95% (for *L. comizo*) of the variation was explained by genetic differentiation within populations (Table 2).

**Table 2:** AMOVAs. Results from the analyses of molecular variance (AMOVAs) conducted independently for each target species. For each species, the highest % of variation explained is presented in bold. Significant *F*_*ST*_ values (*p* < 0.005) indicate significant evidence of population subdivision.

Species	% variation among populations	% variation within populations	*F*_*ST*_
*A. hispanica*	**56.94**	43.06	0.569, *p* < 0.001
*A. occidentale*	**75.58**	24.42	0.756, *p* < 0.001
*A. oligolepis*	**91.91**	8.09	0.919, *p* < 0.001
*I. lemmingii*	37.89	**62.11**	0.379, *p* < 0.001
*I. lusitanicum*	**83.86**	16.14	0.839, *p* < 0.001
*I. almacai*	24.29	**75.71**	0.243, *p* = 0.002
*S. carolitertii*	**71.28**	28.72	0.713, *p* < 0.001
*S. aradensis*	**81.85**	18.15	0.819, *p* < 0.001
*S. pyrenaicus*	**71.88**	28.12	0.719, *p* < 0.001
*S. torgalensis*^a^	–	–	–
*L. bocagei*	**50.65**	49.35	0.507, *p* < 0.001
*L. comizo*	1.05	**98.95**	0.011, *p* = 0.139
*L. sclateri*	9.31	**90.69**	0.093, *p* = 0.001
*P. willkommii*	5.73	**94.27**	0.057, *p* = 0.003
*P. duriense*	45.22	**54.78**	0.452, *p* < 0.001
*P. polylepis*	46.74	**53.26**	0.467, *p* < 0.001

**Notes.**

a AMOVA was not conducted for this species since it has only one population.

### Genetic diversity determinants

Preliminary linear regressions retrieved no outliers when using *h* as the dependent variable. Contrastingly, when using the other genetic diversity indices six outlier populations were detected: *S. carolitertii* from Mondego-Ceira (for *π* only), *S. pyrenaicus* from Tagus-Erges (for MNPD only), and *S. pyrenaicus* from Tagus-Grande da Pipa, *S. pyrenaicus* from Sado-São Martinho, *I. lusitanicum* from Sado-São Martinho and *A. hispanica* from Guadiana-Ardila (for both *π* and MNPD). These populations show a higher number of point mutations, probably due to an admixture of individuals from distinct haplogroups, and those differences between the sequences are reflected in the higher values of *π* and MNPD obtained.

Linear regressions between selected independent variables and *h* values showed that “species maximum size,” “inter-basin connectivity,” “species range,” “latitude,” “migratory behaviour” and “species” had a significant effect on the diversity of populations (Table 3). The independent variables “drainage area” and “hydrological regime” had no significant effect on haplotype diversity (Table 3). After the removal of the outliers, linear regressions using *π* and MNPD as dependent variables retrieved the same pattern (Table 3).

**Table 3:** Correlation coefficients. Regression correlation coefficients (RCC) and their respective *p*-values obtained for the linear regressions between the dependent variables haplotype diversity (*h*), nucleotide diversity (*π*) and mean number of pairwise differences (MNPD) and eight independent variables.

	Dependent variables					
	*h*		*π*		MNPD	
Independent variable	RCC	*p*	RCC	*p*	RCC	*p*
Species	0.516	**<0.001**	0.385	**0.018**	0.485	**<0.001**
Species maximum size	−0.374	**<0.001**	−0.348	**<0.001**	−0.341	**<0.001**
Inter-basin connectivity	0.239	**0.001**	0.244	**0.001**	0.182	**0.001**
Species range	0.193	**0.008**	0.145	**0.050**	0.172	**0.020**
Latitude	0.187	**0.010**	0.226	**0.002**	0.186	**0.012**
Migratory behaviour	0.167	**0.022**	0.154	**0.037**	0.143	**0.054**
Drainage Area	0.113	0.122	0.090	0.227	0.067	0.371
Hydrological regime	0.002	0.978	0.089	0.233	0.078	0.292

Regarding the effect on the *h* index, the inclusion of all independent variables (excluding “species”) in the linear regression, using a stepwise method, indicated that the best fit model only includes “species maximum size,” “inter-basin connectivity” and “latitude.” Non-significant predictor variables “range” and “migratory behaviour” were excluded from the analysis (Table 4). This model explains 26.7% of the haplotype diversity variance (Table 4). The inclusion of the variable “species” in a hierarchical regression analysis had a significant effect, increasing the explained variance to 32.3% (Table 4).

**Table 4:** Hierarchical regression models. Results of the different hierarchical regression models between selected independent variables and the three genetic diversity indices (haplotype diversity, *h*; nucleotide diversity, *π*; and mean number of pairwise differences, MNPD) as dependent variables. For each measure of genetic diversity a series of hierarchical models were fitted based on four key predictor variables (species maximum size, MS; inter-basin connectivity, IBC; latitude, L; and species, S). Four other measured variables were not included because they were shown to be individually unimportant (see Methods). For each model we present adjusted *R*^2^ (coefficient of determination), Δ*R* (*R*^2^ change), test statistics (*F* test statistics) and *p*-values.

	Variables included	Adjusted *R*^2^	Δ*R*	Test statistics	*p*
**Dependent variable: *h***					
Model I	MS	0.135	0.135	*F*_(1,186)_ = 30.214	<0.001
Model II	MS, IBC	0.231	0.096	*F*_(1,186)_ = 24.126	<0.001
Model III	MS, IBC, L	0.267	0.036	*F*_(1,184)_ = 10.139	0.002
Model IV	MS, IBC, L, S	0.323	0.056	*F*_(14,170)_ = 2.087	0.015
**Dependent variable: *π***					
Model I	MS	0.116	0.116	*F*_(1,181)_ = 24.896	<0.001
Model II	MS, IBC	0.213	0.097	*F*_(1,180)_ = 23.288	<0.001
Model III	MS, IBC, L	0.269	0.053	*F*_(1,179)_ = 14.762	<0.001
Model IV	MS, IBC, L, S	0.337	0.068	*F*_(14,165)_ = 2.322	0.006
**Dependent variable: MNPD**					
Model I	MS	0.111	0.058	*F*_(1,181)_ = 23.744	<0.001
Model II	MS, IBC	0.171	0.060	*F*_(1,180)_ = 14.013	<0.001
Model III	MS, IBC, L	0.208	0.037	*F*_(1,179)_ = 9.557	0.002
Model IV	MS, IBC, L, S	0.267	0.059	*F*_(14,165)_ = 2.015	0.019

When applying the same stepwise procedure to the remaining two genetic diversity indices, the results from the linear regressions showed a similar pattern: the same variables were included in the model (“species maximum size,” “inter-basin connectivity,” “latitude” and “species”) and the best fit model explained 33.7% and 26.7% of the variance, respectively, for *π* and MNPD (Table 4).

Concerning the maximum size attained by the species, the results showed a negative correlation with all three genetic diversity indices (Table 3). Regarding “inter-basin connectivity,” the results indicated that populations occurring in sub-basins connected with other water bodies have higher genetic diversity (mean *h* = 0.413 ± 0.306, *N* = 135; mean *π* = 0.00121 ± 0.00120, *N* = 131; mean MNPD = 0.831 ± 0.856, *N* = 131) than those occurring in unconnected river basins (mean *h* = 0.252 ± 0.267, *N* = 53; mean *π* = 0.000606 ± 0.000766, *N* = 52; mean MNPD = 0.508 ± 0.614, *N* = 52). Finally, regarding “latitude,” southern populations exhibited higher levels of haplotype diversity and mean number of pairwise differences (mean *h* = 0.417 ± 0.292, *N* = 108; mean *π* = 0.00126 ± 0.0.00119, *N* = 104; mean MNPD = 0.871 ± 0.830, *N* = 103) than northern populations (mean *h* = 0.302 ± 0.308, *N* = 80; mean *π* = 0.000749 ± 0.000974, *N* = 79; mean MNPD = 0.570 ± 0.747, *N* = 80), a tendency which was already evident from the diversity mapping depicted for the National Genetic Atlas (Fig. 2).

It is worth mentioning that the variable “species” was significantly correlated with the three genetic diversity indices (Table 3) and was included in the model which best explains the observed variance for all of the genetic diversity indices (Table 4). The inclusion of this variable in the best fit models highlights the influence of species intrinsic idiosyncrasies other than those tested explicitly herein (migratory behaviour, maximum size and range). Since it is not possible, with the present dataset, to disentangle which intrinsic traits influence genetic diversity the most, we further analysed which of the extrinsic factors (“drainage area,” “inter-basin connectivity,” “latitude” and “hydrological regime”) were determinant for the genetic diversity pattern observed for each species. The results presented in Table 5 show that the environmental variables tested have no influence on the genetic diversity for most of the species, except for four species (*L. bocagei, P. polylepis, S. pyrenaicus* and *I. lusitanicum*).

**Table 5:** Non-parametric tests for all the species. Results of the non-parametric tests (Spearma’s *ρ* and Mann–Whitney’s *U*) conducted separately to each species to test the correlation between the genetic diversity indices (*h*, *π* and MNPD) and the independent variables “drainage area,” “inter-basin connectivity,” “hydrological regime” and “latitude.” All tests were bilateral and *p*-values were Bonferrori corrected for multiple comparisons. Significant *p*-values (*p* < 0.005) are highlighted in bold and the direction of the Mann–Whitney’s *U* tests was indicated: the group (unconnected vs. connected, temporary vs permanent, and southern vs northern) with the highest median was shaded in grey. Analyses were not conducted for species with less than 5 populations (marked with a “*” symbol). In some cases (marked as “constant”), all the populations of a species showed the same values for a given categorical variable (e.g., all were classified as southern regarding “latitude”).

	Drainage area (DA)		Inter-basin connectivity (IBC)			Hydrological regime (HR)			Latitude (L)		
Species (Nb. populations)	Spearman *ρ*	*p*	U		*p*	U		*p*	U		*p*
			Unconnected	Connected		Temporary	Permanent		Southern	Northern	
**Dependent variable: *h***											
*L. bocagei* (*N* = 39)	0.040	0.808	128		0.578	168		0.988	97		**0.008**
					
*A. oligolepis* (*N* = 26)	−0.169	0.409	88		0.698	34		1.00	35		0.560
					
*P. duriense* (*N* = 14)	0.465	0.094	29		0.240	constant			constant		
	
*P. polylepis* (*N* = 16)	0.257	0.337	constant			20		0.441	2		**0.002**
			
*P. willkommii* (*N* = 6)	−0.543	0.266	constant			constant			constant		
*S. carolitertii* (*N* = 21)	0.114	0.623	69		0.149	13.5		0.667	13.5		0.667
					
*S. pyrenaicus* (*N* = 18)	0.326	0.186	55.5		**0.019**	51		0.075	constant		
			
*S. torgalensis* (*N* = 1)	*		*			*			*		
*S. aradensis* (*N* = 5)	0.447	0.450	constant			constant			constant		
*L. comizo* (*N* = 6)	0.395	0.439	constant			1		0.667	constant		
	
*L. sclateri* (*N* = 9)	0.402	0.284	12		0.730	constant			constant		
	
*I. lusitanicum* (*N* = 14)	0.804	**0.001**	45		**<0.001**	37		0.060	constant		
			
*I. almacai* (*N* = 2)	*		*			*			*		
*I. lemmingii* (*N* = 3)	*		*			*			*		
*A. occidentale* (*N* = 3)	*		*			*			*		
*A. hispanica* (*N* = 5)	0.300	0.624	constant			constant			constant		
**Dependent variable:** *π*											
*L. bocagei* (*N* = 39)	0.074	0.074	133		0.462	154		0.670	94		**0.006**
					
*A. oligolepis* (*N* = 26)	−0.088	0.669	94		0.482	33		0.940	31		0.389
					
*P. duriense* (*N* = 14)	0.251	0.387	30.5		0142	constant			constant		
	
*P. polylepis* (*N* = 16)	0.257	0.337	constant			18		0.320	1		**<0.001**
			
*P. willkommii* (*N* = 6)	−0.486	0.329	constant			constant			constant		
*S. carolitertii* (*N* = 21)	0.226	0.338	63		0.183	12.5		0.700	13.5		0.667
					
*S. pyrenaicus* (*N* = 18)	0.094	0.711	51		0.075	49		0.117	constant		
			
*S. torgalensis* (*N* = 1)	*		*			*			*		
*S. aradensis* (*N* = 5)	0.447	0.450	constant			constant			constant		
*L. comizo* (*N* = 6)	0.339	0.510	constant			1		0.667	constant		
	
*L. sclateri* (*N* = 9)	0.485	0.185	14		0.413	constant			constant		
	
*I. lusitanicum* (*N* = 14)	0.453	0.120	45		**<0.001**	30		0.364	constant		
			
*I. almacai* (*N* = 2)	*		*			*			*		
*I. lemmingii* (*N* = 3)	*		*			*			*		
*A. occidentale* (*N* = 3)	*		*			*			*		
*A. hispanica* (*N* = 5)	0.1	0.873	constant			constant			constant		
**Dependent variable: MNPD**											
*L. bocagei* (*N* = 39)	0.074	0.656	133		0.462	154		0.670	94		**0.006**
					
*A. oligolepis* (*N* = 26)	−0.088	0.669	94		0.484	33		0.940	31		0.389
					
*P. duriense* (*N* = 14)	0.251	0.387	30.5		0.142	constant			constant		
	
*P. polylepis* (*N* = 16)	0.257	0.337	constant			18		0.320	1		**<0.001**
			
*P. willkommii* (*N* = 6)	−0.486	0.329	constant			constant			constant		
*S. carolitertii* (*N* = 21)	0.185	0.422	70		0.128	13.5		0.667	13.5		0.667
					
*S. pyrenaicus* (*N* = 18)	−0.125	0.621	41		0.443	51		0.075	constant		
			
*S. torgalensis* (*N* = 1)	*		*			*			*		
*S. aradensis* (*N* = 5)	0.200	0.747	constant			constant			constant		
*L. comizo* (*N* = 6)	0.395	0.439	constant			1		0.667	constant		
	
*L. sclateri* (*N* = 9)	0.485	0.185	14		0.413	constant			constant		
		
*I. lusitanicum* (*N* = 14)	0.453	0.120	45		**<0.001**	30		0.364	constant		
			
*I. almacai* (*N* = 2)	*		*			*			*		
*I. lemmingii* (*N* = 3)	*		*			*			*		
*A. occidentale* (*N* = 3)	*		*			*			*		
*A. hispanica* (*N* = 5)	0.1	0.873	constant			constant			constant		

More specifically, all of the genetic diversity indices were significantly correlated with “inter-basin connectivity” for *I. lusitanicum* and with “latitude” for *L. bocagei* and *P. polylepis* (Table 5). The haplotype diversity obtained was also significantly correlated with “inter-basin connectivity” for *S. pyrenaicus* and with “drainage area” for *I. lusitanicum* (Table 5).

## Discussion

The sampling of a broad number of populations throughout the distribution range of cyprinid species in Portugal allowed for the publication of the first National Genetic Atlas of native cyprinid ichthyofauna (available online at www.fishatlas.net). The analysis of the genetic diversity variation highlighted differences among populations within species and also differences between species with identical distribution areas and threat categories. In general, the percentage of private haplotypes and the average values of genetic diversity *per* population were higher for small sized non-migratory species of the genera *Anaecypris, Achondrostoma, Iberochondrostoma* and *Squalius* than for the larger sized potamodromous *Luciobarbus* and *Pseudochondrostoma* species, raising the hypothesis of species intrinsic determinants of genetic diversity. Also, the analyses of molecular variance revealed that for some species most of the variance could be attributed to differences among populations, while in others to differentiation within populations. The spatial distribution of the genetic diversity was undoubtedly distinct for co-occurring species, but what are the underlying causes of such distinct patterns?

Concerning freshwater fish, the distribution of the genetic diversity on the landscape may be determined by (1) historical geological/climatic processes (e.g., drainage rearrangements, persistent climatic gradients, glaciations), (2) species intrinsic traits (e.g., body size, fecundity, dispersal ability), and (3) landscape features, including human-mediated habitat changes (e.g., damming, river dewatering, destruction of optimal habitats) (Osborne et al., 2014). Determinants of genetic variation have been, however, difficult to identify and/or disentangle (Husemann et al., 2012; Leffler et al., 2012).

### Historical determinants of genetic diversity

The present study, targeting multiple co-distributed species of primary freshwater fish, assessed the relative role of historical *versus* contemporary factors affecting genetic diversity. Indeed, the origin of Iberian lineages of *Anaecypris*, ex-*Chondrostoma, Luciobarbus* and *Squalius* dates back to the Miocene, around 19–7.7 Mya (Levy, Doadrio & Almada, 2008; Gante, 2011). Speciation within these genera and subsequent diversification must have occurred through the same available connections between paleobasins until the Pleistocene-Holocene, when the current hydrographical network became established (Sousa-Santos, Collares-Pereira & Almada, 2007; Gante et al., 2009; Almada & Sousa-Santos, 2010; Sousa-Santos et al., 2014a; Sousa-Santos et al., 2014b; Mesquita et al., 2005). As such, if populations responded identically to landscape rearrangements and climatic conditions through time, one would expect the patterns of genetic diversity to be similar for co-occurring species, despite their intrinsic traits.

It is known that during the last glacial maximum (LGM, 0.018 Mya) the ice sheet reached the central part of the Portuguese territory, presumably as far as the Tagus River (Fig. 1A, Dias et al., 2000). Previous phylogenetic data, obtained with a calibrated molecular marker (cytochrome *b*), indicates that, at the time of the LGM, all contemporary species were already differentiated (Levy, Doadrio & Almada, 2008; Gante, 2011). Additionally, the extirpation of fish populations at northern latitudes and the persistence of Iberian fish species in southern refugia throughout the Quaternary had already been reported by several authors (e.g., Mesquita et al., 2005; Gante et al., 2009; Almada & Sousa-Santos, 2010; Araguas et al., 2013; Sousa-Santos et al., 2014b; Perea & Doadrio, 2015). Thus, as a consequence of the LGM, species inhabiting northern river basins should exhibit similar low levels of genetic diversity. Data presented in this paper shows that although this is true for *L. bocagei*, it is far from being a generalized pattern among northerly distributed species. The relatively high levels of genetic diversity observed in *A. oligolepis, P. duriense* and *S. carolitertii* could be explained by posterior recolonizations of northern streams by migrants from southern refugia, as suggested for many aquatic species (e.g., Hewitt, 2004; Gómez & Lunt, 2007; Provan & Bennett, 2008; Roselló & Morales, 2010; Oberdoff et al., 2011). However, this hypothesis is not plausible for the target species since at the time of the LGM most of the connections between river basins had already ceased (Pais et al., 2012). Since differences between species with identical distribution areas were detected for the three genetic diversity indices used, one must postulate than contemporary determinants of genetic diversity must have played a more significant role than historical ones.

### Potential contemporary determinants of genetic diversity

As referred above, species intrinsic traits and landscape features may influence current levels of genetic diversity. Habitat loss and fragmentation, for instance, may result in declining population sizes and genetic diversity depletion, ultimately leading to local extinctions (Frankham, 1995; Spielman, Brook & Frankham, 2004; Ewers & Didham, 2006; Lowe & Allendorf, 2010). Husemann et al. (2012) found that frequent local extinction and re-colonization cycles in seasonal pools may even obscure historical signatures in the genetic patterns of some North American cyprinids as a result of repeated bottlenecks and population expansions. Demographic and genetic changes will impact species differently, according to their dispersal ability, life-history characteristics and habitat requirements (Faulks, Gilligan & Beheregaray, 2011).

Ecological traits such as tolerance to stagnant disconnected summer pools (Husemann et al., 2012), preference for flowing headwaters (Faulks, Gilligan & Beheregaray, 2011; Buonerba et al., 2015) or reproductive strategies (benthic vs. pelagic spawning, Osborne et al., 2014) were pointed out as determinants of genetic diversity in a vast array of freshwater fish. Our results showed that there are intrinsic characteristics (maximum size attained) and environmental characteristics (inter-basin connectivity and latitude) that are clearly determinant for genetic differences between populations. The variable “species” includes additional intrinsic characteristics other than those considered explicitly that could be causing the observed patterns of genetic variation. The relevance of this component is underlined by its significant correlation with genetic diversity indices and by its inclusion in the best fit model (together with “species maximum size,” “inter-basin connectivity” and “latitude” explained 26.7%–33.7% of the variance). The causes explaining the remaining 66.3%–73.3% of the genetic diversity variance remained currently unexplained and should ideally be addressed in future studies.

#### (i) Species intrinsic traits

The species maximum size was negatively correlated with genetic diversity, a feature that was already reported for several taxa (e.g., Romiguier et al., 2014). Body size is a good predictor of maturation age and egg size (Moyle & Cech, 2004): small sized fish are precocious spawners and lay more eggs *per* batch (*r*-strategists) than larger sized fish that typically mature later and produce a smaller number of eggs (*k*-strategists). As a consequence, in disturbed environments that impose shorter than normal life spans, it is expected that small sized species would be favoured as they will most likely leave more progeny than larger species and will presumably be less prone to genetic depletion due to inbreeding and genetic drift (more intense in populations with small effective sizes, Gilpin & Soulé, 1986; Vrijenhoek, 1994). As argued by Romiguier et al. (2014), the demographic impact of environmental perturbations will depend on the species life-history strategy: typically genetic diversity levels will be higher *r*-strategists than in *k*-strategists irrespective of their current demography which, according to the authors, also explains why *r*-strategists might be in risk of extinction without any warning genetic signal (see “*Implications for conservation”* below).

#### (ii) Environmental characteristics

Drainage area and hydrological regime (permanent vs. temporary) were not significantly correlated with genetic diversity, while connectivity between sub-basins and latitude had a significant effect.

The results show that populations occurring in sub-basins connected with other water bodies had higher genetic diversity than those occurring in unconnected river basins. As the drainage had no significant effect on the genetic diversity of the populations inhabiting them, the observed effect of the inter-basin connectivity may be related to (1) the possibility of inter-population gene exchange or (2) historical features. This later explanation relates to the fact that the colonization of Iberia by the ancestral lineages of primary freshwater fish seems to have followed a westward path (from the Pyrenees to the margins), with the major basins as vehicles for colonizers and playing a crucial role in the radiation of these species throughout the Peninsula (e.g., Gante et al., 2009; Almada & Sousa-Santos, 2010; Perea & Doadrio, 2014). As a consequence, the unconnected river basins that showed lower levels of genetic diversity are precisely the small coastal streams from the west and southwest margins of Iberia which received their colonizers through past connections with the wider and dendritic river basins. Hence, this pattern may be a result of a decrease in genetic diversity along routes of colonization (Taberlet et al., 1998).

Future studies should also address connectivity at an intrabasin scale, by quantifying the number of unsurmountable barriers preventing gene flow. Predictions point to lower global levels of genetic diversity due to fragmentation and population declines expected to occur in highly impounded river basins (e.g., Alò & Turner, 2005; Wofford, Gresswell & Banks, 2005; Raeymaekers et al., 2008; Blanchet et al., 2010).

Genetic diversity also seems to be related with latitude: southern populations show higher levels of haplotype diversity and mean number of pairwise differences than northern populations. This pattern may be the result of a higher impact of glaciations in northern populations, and *in situ* survival in the southernmost populations, far from the glaciers (e.g., Hoagstrom & Berry, 2006; Abellán & Svenning, 2014; Osborne et al., 2014). The successive cycles of expansion–contraction of glacial ice sheets must have played an extremely important role in shaping the distribution of *taxa* and in inducing population declines and local extinctions (Rowe et al., 2004). However, if glaciations were the main driver of genetic diversity depletion in northern populations, one should expect that all those populations inhabiting northern rivers (where the effect of the LGM was more effective, as described by Dias et al., 2000) would exhibit low levels of genetic diversity independently of the species considered, which was not the case. This expectation would fail if species inhabiting northern rivers had distinct tolerances to low water temperatures and, consequently, showed differential survival during glacial periods. Additionally, the effect of temperature should not be ruled out since it is known that high water temperatures may induce genetic damage and errors in DNA replication (Gillooly et al., 2005), which may eventually promote higher levels of diversity in the populations inhabiting warmer southern rivers.

The fragmentation of populations imposed annually by extreme droughts (Prenda & Gallardo, 1996; Pires, Cowx & Coelho, 1999; Magalhães et al., 2002) could also be pointed out as one of the determinants for higher genetic diversity levels of southern populations, by imposing higher drift and lineage sorting effects. If true, one would expect that populations inhabiting temporary rivers show distinct genetic diversity from those inhabiting permanent rivers. However, our results show that the “hydrological regime” had no significant effect.

Finally, when looking to each isolated species rather than using populations as the comparison units, the results showed that environmental characteristics did not explain the genetic diversity variance for most of the species, with the exception of the potamodromous large sized *L. bocagei* and *P. polylepis* and the smaller sized non-potamodromous *S. pyrenaicus* and *I. lusitanicum*. These species showed higher genetic diversity in larger river basins (*I. lusitanicum*), in southern latitudes (*L. bocagei* and *P. polylepis*) and in connected sub-basins (*S. pyrenaicus* and *I. lusitanicum*). These results are in agreement with the view of Kahilainen, Puurtinen & Kotiaho (2014) regarding the differential influence of environmental characteristics on the genetic diversity of co-occurring species and highlights the need to establish specific rather than generalized management and conservation plans.

In conclusion, as multiple intrinsic and extrinsic drivers may be acting synergistically, future studies should ideally be conducted for each species separately and should adopt sampling procedures that would allow for an exhaustive collection of data concerning habitat-, landscape- and species-related variables.

#### Implications for conservation

Different populations of the same species exhibit not only distinct gene pools but also distinct genetic diversity levels, reinforcing the need to preserve them as individual entities and to establish Operational Conservation Units (OTU’s), as defined by Doadrio, Perdices & Machordom (1996).

A high risk of extinction is commonly associated with low levels of genetic diversity and small effective population size (e.g., Blomqvist et al., 2010; McCusker & Bentzen, 2013). As argued by some authors, most *taxa* are not driven to extinction before genetic factors affect them adversely (Van Noordwijk, 1994; Spielman, Brook & Frankham, 2004), thus, endangered species should be closely monitored to assess potential future drops in gene diversity levels. Among *Squalius* species, the critically endangered *S. aradensis* and *S. torgalensis* showed lower haplotype diversities than the remaining *Squalius* species, which are considered to be endangered (*S. pyrenaicus*) and least concerned (*S. carolitertii*). The same occurs among *Iberochondrostoma* and *Achondrostoma* species, with the critically endangered *I. almacai* and *A. occidentale* showing lower haplotype diversity than their less threatened congeneric species *I. lemmingii* (endangered) and *A. oligolepis* (vulnerable). A similar pattern was also detected among *Luciobarbus*, with the endangered *L. comizo* and *L. sclateri* showing lower haplotype diversities than the least concerned *L. bocagei*. Interestingly, however, the results obtained in this study indicate that a higher conservation status is not necessarily synonymous with genetic depletion, as demonstrated by the high haplotype diversity values shown by the critically endangered *A. hispanica* and *I. lusitanicum*. The reverse was also not always observed since non-endangered *L. bocagei, P. polylepis* or *S. carolitertii* showed relatively low levels of haplotype diversity (when compared to their congeneric species). These results highlight the need to focus management and conservation actions on intraspecific genetic data, instead of erroneously concluding that a species with low genetic diversity is more susceptible to extinction than a co-occurring more diversified one. Frequent genetic monitoring is also crucial since it is known that there is a time lag between the action of factors causing genetic change and the change itself, i.e., changes or disturbances that impact populations may not be immediately reflected in genetic metrics (Epps & Keyghobadi, 2015). The combination of long term genetic and demographic surveys of threatened species should be the norm in conservation practices, as suggested by Paz-Vinas et al. (2013).

The dataset produced under the scope of the FISHATLAS project is available to be used by managers, decision-makers and authorities not only in the present context of hydrological resources management aiming to minimize the effects of climate changes, but also for the implementation of conservation and management plans aiming to preserve native Iberian cyprinids. More specifically, these data may allow for the definition of priorities in conservation policies, when choices have to be made concerning which populations of each species must be preserved first, a decision that must take into account the maximization of genetic diversity. Although it is widely recognized that genetic data should be taken into account to draw conservation guidelines and prioritize conservation actions (Frankham, 2010), studies on conservation genetics of Iberian cyprinids are recent (Salgueiro et al., 2003; Robalo et al., 2007; Sousa et al., 2007; Almada & Sousa-Santos, 2010; Sousa et al., 2010; Sousa-Santos et al., 2014a; Sousa-Santos et al., 2014b) and practical applications of their conclusions and suggestions are still scarce. The results presented herein constitute a comprehensive baseline dataset which, supplemented with future monitoring of the observed genetic patterns, will be crucial to support the establishment of conservation priorities, design reserves, signal target populations for *ex situ* conservation, define OUT’s, and propose *ex situ* and *in situ* actions to allow for the long-term survival of endangered species and preservation of their genetic integrity.

## Supplemental Information

10.7717/peerj.1694/supp-1 Table S1 Specimens collected in each river basin/sub-basin Number of specimens collected in each river basin/sub-basin. Legend: CR, Critically endangered; EN, Endangered; VU, Vulnerable; LC, Least concerned; NT, Not Threatened.

10.7717/peerj.1694/supp-2 Table S2 Data matrix Matrix of independent and dependent variables used for statistical analyses.

## Additional Information and Declarations

### Field Study Permissions

The following information was supplied relating to field study approvals (i.e., approving body and any reference numbers): Permits for field work were given by ICNF (176/2010/CAPT and 53/2012/CAPT).

### DNA Deposition

The following information was supplied regarding the deposition of DNA sequences: GenBank accession numbers: KU366823– KU370500.

### Data Availability

The following information was supplied regarding data availability: Besides sequencing data, raw data for the statistical analyses performed are provided in Table S2.
