# Supplementary material for: Broad-scale sampling of primary freshwater fish populations reveals the role of intrinsic traits, inter-basin connectivity, drainage area and latitude on shaping contemporary patterns of genetic diversity
Source: PeerJ. 2016 Feb 29;4:e1694. doi: 10.7717/peerj.1694 (PMC4782715; doi:10.7717/peerj.1694)
Supplement: Table S1 — Number of specimens collected in each river basin/sub-basin. Legend: CR, Critically endangered; EN, Endangered; VU, Vulnerable; LC, Least concerned; NT, Not Threatened. [file peerj-04-1694-s001.docx]

Table S1 – Number of specimens collected in each river basin/sub-basin. Legend: CR – Critically endangered, EN – Endangered, VU – Vulnerable, LC – Least Concerned, NT – Not Threatened.

| Species |  | *Pseudochondrostoma duriense* | *Pseudochondrostoma polylepis* | *Pseudochondrostoma willkommii* | *Squalius carolitertii* | *Squalius pyrenaicus* | *Squalius aradensis* | *Squalius torgalensis* | *Luciobarbus bocagei* | *Luciobarbus comizo* | *Luciobarbus sclateri* | *Iberochondrostoma lusitanicum* | *Iberochondrostoma almacai* | *Iberochondrostoma lemmingii* | *Achondrostoma oligolepis* | *Achondrostoma occidentale* | *Anaecypris hispanica* |
| --- | --- | --- | --- | --- | --- | --- | --- | --- | --- | --- | --- | --- | --- | --- | --- | --- | --- |
| Threat categories |  | LC | LC | VU | LC | EN | CR | CR | LC | EN | EN | CR | CR | EN | VU | CR | CR |
| River Basin /  River Sub-basin | Number in Fig.1a |  |  |  |  |  |  |  |  |  |  |  |  |  |  |  |  |
| Minho | 1 |  |  |  | 18 |  |  |  |  |  |  |  |  |  |  |  |  |
| Âncora | 2 |  |  |  |  |  |  |  |  |  |  |  |  |  | 20 |  |  |
| Cabanas | 3 |  |  |  |  |  |  |  |  |  |  |  |  |  | 20 |  |  |
| Pego | 4 |  |  |  | 18 |  |  |  |  |  |  |  |  |  | 17 |  |  |
| Lima | 5 | 19 |  |  | 21 |  |  |  | 19 |  |  |  |  |  | 21 |  |  |
| Neiva | 6 | 15 |  |  | 31 |  |  |  |  |  |  |  |  |  | 17 |  |  |
| Cávado | 7 | 15 |  |  | 24 |  |  |  | 17 |  |  |  |  |  | 23 |  |  |
| Ave | 8 | 18 |  |  | 20 |  |  |  | 17 |  |  |  |  |  | 17 |  |  |
| Douro - Sousa | 9 | 20 |  |  | 20 |  |  |  | 20 |  |  |  |  |  |  |  |  |
| Douro - Tâmega | 10 | 17 |  |  | 30 |  |  |  | 21 |  |  |  |  |  |  |  |  |
| Douro - Corgo | 11 | 20 |  |  |  |  |  |  | 20 |  |  |  |  |  | 18 |  |  |
| Douro - Tua | 12 | 19 |  |  |  |  |  |  | 18 |  |  |  |  |  | 17 |  |  |
| Douro - Sabor | 13 | 24 |  |  | 17 |  |  |  | 18 |  |  |  |  |  | 24 |  |  |
| Douro - Paiva | 14 | 19 |  |  | 19 |  |  |  | 20 |  |  |  |  |  | 24 |  |  |
| Douro - Távora | 15 | 17 |  |  | 16 |  |  |  | 20 |  |  |  |  |  |  |  |  |
| Douro - Coa | 16 |  |  |  | 18 |  |  |  | 19 |  |  |  |  |  | 19 |  |  |
| Vouga - Caima | 17 | 16 |  |  | 19 |  |  |  | 20 |  |  |  |  |  | 22 |  |  |
| Vouga - Sul | 18 | 18 |  |  | 34 |  |  |  |  |  |  |  |  |  | 17 |  |  |
| Vouga - Mel | 19 |  |  |  |  |  |  |  |  |  |  |  |  |  | 19 |  |  |
| Vouga - Águeda | 20 | 17 |  |  | 19 |  |  |  | 19 |  |  |  |  |  | 19 |  |  |
| Mondego - Mortágua | 21 |  | 21 |  | 15 |  |  |  | 20 |  |  |  |  |  | 20 |  |  |
| Mondego - Dão | 22 |  | 17 |  | 19 |  |  |  | 18 |  |  |  |  |  | 19 |  |  |
| Mondego - Arunca | 23 |  | 15 |  |  |  |  |  | 17 |  |  |  |  |  | 18 |  |  |
| Mondego - Corvo | 24 |  | 20 |  | 18 |  |  |  | 15 |  |  |  |  |  | 17 |  |  |
| Mondego - Ceira | 25 |  |  |  | 15 |  |  |  | 21 |  |  |  |  |  | 17 |  |  |
| Mondego - Alva | 26 |  | 18 |  | 19 |  |  |  | 17 |  |  |  |  |  | 20 |  |  |
| Lis | 27 |  |  |  |  |  |  |  | 17 |  |  |  |  |  |  |  |  |
| São Pedro | 28 |  |  |  |  |  |  |  |  |  |  |  |  |  | 16 |  |  |
| Alcoa | 29 |  |  |  | 20 |  |  |  | 19 |  |  |  |  |  | 19 |  |  |
| Tornada | 30 |  |  |  |  |  |  |  |  |  |  |  |  |  |  |  |  |
| Real | 31 |  |  |  |  |  |  |  |  |  |  |  |  |  | 20 |  |  |
| Alcabrichel | 32 |  |  |  |  |  |  |  |  |  |  |  |  |  |  | 23 |  |
| Sizandro | 33 |  |  |  |  |  |  |  |  |  |  |  |  |  |  | 27 |  |
| Safarujo | 34 |  |  |  |  |  |  |  |  |  |  |  |  |  |  | 22 |  |
| Lizandro | 35 |  |  |  |  | 20 |  |  | 15 |  |  | 23 |  |  |  |  |  |
| Samarra | 36 |  |  |  |  | 21 |  |  |  |  |  | 19 |  |  |  |  |  |
| Colares | 37 |  |  |  |  | 21 |  |  | 18 |  |  | 18 |  |  |  |  |  |
| Barcarena | 38 |  |  |  |  |  |  |  |  |  |  | 17 |  |  |  |  |  |
| Jamor | 39 |  |  |  |  | 20 |  |  |  |  |  | 17 |  |  |  |  |  |
| Tagus - Erges | 40 |  | 18 |  |  | 18 |  |  | 16 |  |  |  |  |  |  |  |  |
| Tagus - Ponsul | 41 |  | 15 |  |  | 17 |  |  |  |  |  |  |  |  |  |  |  |
| Tagus - Ocreza | 42 |  | 18 |  |  |  |  |  | 22 | 15 |  |  |  |  |  |  |  |
| Tagus - Zezere | 43 |  |  |  |  |  |  |  |  |  |  |  |  |  |  |  |  |
| Tagus - Zêzere Nabão | 44 |  | 20 |  |  |  |  |  | 16 |  |  |  |  |  | 21 |  |  |
| Tagus - Zezere Sertã | 45 |  |  |  |  |  |  |  | 15 |  |  |  |  |  |  |  |  |
| Tagus - Almonda | 46 |  | 20 |  |  | 16 |  |  | 17 |  |  |  |  |  |  |  |  |
| Tagus - Alviela | 47 |  | 17 |  |  |  |  |  | 19 |  |  | 17 |  |  |  |  |  |
| Tagus - Maior | 48 |  |  |  |  |  |  |  |  |  |  | 50 |  |  |  |  |  |
| Tagus - Ota | 49 |  |  |  |  | 20 |  |  | 15 |  |  | 19 |  |  |  |  |  |
| Tagus - Grande da pipa | 50 |  |  |  |  | 20 |  |  |  |  |  | 20 |  |  |  |  |  |
| Tagus - Trancão | 51 |  |  |  |  |  |  |  |  |  |  | 19 |  |  |  |  |  |
| Tagus - Sever | 52 |  | 16 |  |  |  |  |  |  |  |  |  |  |  |  |  |  |
| Tagus - Nisa | 53 |  | 18 |  |  |  |  |  | 15 |  |  |  |  |  |  |  |  |
| Tagus - Muge | 54 |  | 18 |  |  | 15 |  |  | 20 |  |  |  |  |  |  |  |  |
| Tagus - Sorraia | 55 |  | 21 |  |  |  |  |  |  |  |  |  |  |  |  |  |  |
| Tagus - Coina | 56 |  |  |  |  |  |  |  |  |  |  | 20 |  |  |  |  |  |
| Sado - Roxo | 57 |  |  |  |  |  |  |  | 19 |  |  |  |  |  |  |  |  |
| Sado - Odivelas | 58 |  |  |  |  | 20 |  |  | 20 |  |  | 20 |  |  |  |  |  |
| Sado - Xarrama | 59 |  |  |  |  |  |  |  | 19 |  |  |  |  |  |  |  |  |
| Sado - Alcaçovas | 60 |  |  |  |  |  |  |  |  |  |  |  |  |  |  |  |  |
| Sado - S.Martinho | 61 |  |  |  |  | 20 |  |  | 20 |  |  | 18 |  |  |  |  |  |
| Sado - Marateca | 62 |  |  |  |  |  |  |  | 19 |  |  |  |  |  |  |  |  |
| Sado - Campilhas | 63 |  | 16 |  |  | 19 |  |  | 20 |  |  |  |  |  |  |  |  |
| Sado - Corona | 64 |  |  |  |  |  |  |  | 19 |  |  |  |  |  |  |  |  |
| Sado - Grândola | 65 |  |  |  |  |  |  |  |  |  |  | 20 |  |  |  |  |  |
| Mira | 66 |  |  |  |  |  |  | 21 |  |  | 16 |  | 20 |  |  |  |  |
| Seixe | 67 |  |  |  |  |  | 20 |  |  |  | 18 |  |  |  |  |  |  |
| Aljezur | 68 |  |  |  |  |  | 20 |  |  |  |  |  |  |  |  |  |  |
| Alvor | 69 |  |  |  |  |  | 18 |  |  |  |  |  |  |  |  |  |  |
| Arade | 70 |  |  |  |  |  | 20 |  |  |  | 17 |  | 20 |  |  |  |  |
| Quarteira | 71 |  |  |  |  |  | 20 |  |  |  |  |  |  |  |  |  |  |
| Gilão | 72 |  |  |  |  | 17 |  |  |  |  | 15 |  |  |  |  |  |  |
| Guadiana - Ardila | 73 |  |  |  |  |  |  |  |  | 35 | 27 |  |  |  |  |  | 22 |
| Guadiana - Chança | 74 |  |  | 20 |  |  |  |  |  | 23 | 33 |  |  | 18 |  |  | 21 |
| Guadiana - Caia | 75 |  |  | 18 |  | 19 |  |  |  |  |  |  |  |  |  |  |  |
| Guadiana - Degebe | 76 |  |  | 18 |  |  |  |  |  | 16 |  |  |  | 19 |  |  |  |
| Guadiana - Cobres | 77 |  |  | 19 |  | 19 |  |  |  | 18 |  |  |  |  |  |  |  |
| Guadiana - Oeiras | 78 |  |  | 19 |  | 23 |  |  |  |  | 22 |  |  |  |  |  |  |
| Guadiana - Vascão | 79 |  |  | 19 |  |  |  |  |  | 23 | 36 |  |  |  |  |  | 23 |
| Guadiana - Odeleite | 80 |  |  |  |  | 18 |  |  |  |  | 25 |  |  | 20 |  |  | 20 |
| Guadiana - Foupana | 80a |  |  |  |  |  |  |  |  |  |  |  |  |  |  |  | 23 |
