# Supplementary material for: Broad-scale sampling of primary freshwater fish populations reveals the role of intrinsic traits, inter-basin connectivity, drainage area and latitude on shaping contemporary patterns of genetic diversity
Source: PeerJ. 2016 Feb 29;4:e1694. doi: 10.7717/peerj.1694 (PMC4782715; doi:10.7717/peerj.1694)
Supplement: Table S2 — Matrix of independent and dependent variables used for statistical analyses. [file peerj-04-1694-s002.docx]

Table S2 – Matrix of independent and dependent variables used for statistical analyses.

|  | **Independent variables (intrinsic)** | | | | **Independent variables (extrinsic)** | | | | **Dependent variables** | | |
| --- | --- | --- | --- | --- | --- | --- | --- | --- | --- | --- | --- |
| **Population** | **Species** | **Species**  **max. Size (cm)** | **Migratory**  **behaviour** | **Range** | **Drainage**  **area (km2)** | **Hydrology** | **Latitude** | **Inter-basin**  **connectivity** | **Haplotype**  **diversity** | **Nucleotide**  **diversity** | **MNPD** |
| Lima | *L. bocagei* | 100 | potamodromous | wide | 1166.74 | permanent | north | unconnected | 0.456 | 0.00063 | 0.456 |
| Cávado | *L. bocagei* | 100 | potamodromous | wide | 1609.97 | permanent | north | unconnected | 0.000 | 0.00000 | 0.000 |
| Ave | *L. bocagei* | 100 | potamodromous | wide | 1390.34 | permanent | north | unconnected | 0.000 | 0.00000 | 0.000 |
| Douro-Sousa | *L. bocagei* | 100 | potamodromous | wide | 557.22 | permanent | north | connected | 0.000 | 0.00000 | 0.000 |
| Douro-Tâmega | *L. bocagei* | 100 | potamodromous | wide | 2643.24 | permanent | north | connected | 0.095 | 0.00013 | 0.095 |
| Douro-Corgo | *L. bocagei* | 100 | potamodromous | wide | 469.39 | permanent | north | connected | 0.000 | 0.00000 | 0.000 |
| Douro-Tua | *L. bocagei* | 100 | potamodromous | wide | 3122.20 | permanent | north | connected | 0.000 | 0.00000 | 0.000 |
| Douro-Sabor | *L. bocagei* | 100 | potamodromous | wide | 2377.22 | permanent | north | connected | 0.000 | 0.00000 | 0.000 |
| Douro-Paiva | *L. bocagei* | 100 | potamodromous | wide | 790.37 | permanent | north | connected | 0.000 | 0.00000 | 0.000 |
| Douro-Távora | *L. bocagei* | 100 | potamodromous | wide | 531.87 | permanent | north | connected | 0.000 | 0.00000 | 0.000 |
| Douro-Coa | *L. bocagei* | 100 | potamodromous | wide | 2520.69 | permanent | north | connected | 0.000 | 0.00000 | 0.000 |
| Vouga-Caima | *L. bocagei* | 100 | potamodromous | wide | 211.06 | permanent | north | connected | 0.000 | 0.00000 | 0.000 |
| Vouga-Águeda | *L. bocagei* | 100 | potamodromous | wide | 385.19 | permanent | north | connected | 0.000 | 0.00000 | 0.000 |
| Mondego-Mortágua | *L. bocagei* | 100 | potamodromous | wide | 198.97 | permanent | north | connected | 0.195 | 0.00028 | 0.200 |
| Mondego-Dão | *L. bocagei* | 100 | potamodromous | wide | 1127.77 | permanent | north | connected | 0.000 | 0.00000 | 0.000 |
| Mondego-Arunca | *L. bocagei* | 100 | potamodromous | wide | 729.47 | permanent | north | connected | 0.118 | 0.00016 | 0.118 |
| Mondego-Corvo | *L. bocagei* | 100 | potamodromous | wide | 228.97 | permanent | north | connected | 0.000 | 0.00000 | 0.000 |
| Mondego-Ceira | *L. bocagei* | 100 | potamodromous | wide | 503.19 | permanent | north | connected | 0.381 | 0.00053 | 0.381 |
| Mondego-Alva | *L. bocagei* | 100 | potamodromous | wide | 707.63 | permanent | north | connected | 0.000 | 0.00000 | 0.000 |
| Lis | *L. bocagei* | 100 | potamodromous | wide | 902.89 | temporary | south | unconnected | 0.529 | 0.00074 | 0.529 |
| Alcoa | *L. bocagei* | 100 | potamodromous | wide | 421.19 | temporary | south | unconnected | 0.000 | 0.00000 | 0.000 |
| Lizandro | *L. bocagei* | 100 | potamodromous | wide | 167.58 | temporary | south | unconnected | 0.000 | 0.00000 | 0.000 |
| Colares | *L. bocagei* | 100 | potamodromous | wide | 49.65 | temporary | south | unconnected | 0.000 | 0.00000 | 0.000 |
| Tejo-Erges | *L. bocagei* | 100 | potamodromous | wide | 592.83 | permanent | south | connected | 0.667 | 0.00151 | 1.083 |
| Tejo-Ocreza | *L. bocagei* | 100 | potamodromous | wide | 1429.42 | permanent | south | connected | 0.472 | 0.00073 | 0.528 |
| Tejo-Zêzere Nabão | *L. bocagei* | 100 | potamodromous | wide | 1016.84 | permanent | south | connected | 0.125 | 0.00035 | 0.250 |
| Tejo-Zezere Sertã | *L. bocagei* | 100 | potamodromous | wide | 331.01 | permanent | south | connected | 0.648 | 0.00109 | 0.781 |
| Tejo-Almonda | *L. bocagei* | 100 | potamodromous | wide | 237.16 | permanent | south | connected | 0.404 | 0.00059 | 0.426 |
| Tejo-Alviela | *L. bocagei* | 100 | potamodromous | wide | 371.78 | permanent | south | connected | 0.351 | 0.00049 | 0.351 |
| Tejo-Ota | *L. bocagei* | 100 | potamodromous | wide | 287.42 | permanent | south | connected | 0.686 | 0.00143 | 1.029 |
| Tejo-Nisa | *L. bocagei* | 100 | potamodromous | wide | 264.16 | temporary | south | connected | 0.133 | 0.00019 | 0.133 |
| Tejo-Muge | *L. bocagei* | 100 | potamodromous | wide | 703.14 | temporary | south | connected | 0.268 | 0.00037 | 0.268 |
| Sado-Roxo | *L. bocagei* | 100 | potamodromous | wide | 688.76 | temporary | south | connected | 0.526 | 0.00219 | 1.579 |
| Sado-Odivelas | *L. bocagei* | 100 | potamodromous | wide | 737.21 | temporary | south | connected | 0.395 | 0.00165 | 1.184 |
| Sado-Xarrama | *L. bocagei* | 100 | potamodromous | wide | 523.46 | temporary | south | connected | 0.000 | 0.00000 | 0.000 |
| Sado-S.Martinho | *L. bocagei* | 100 | potamodromous | wide | 246.39 | temporary | south | connected | 0.000 | 0.00000 | 0.000 |
| Sado-Marateca | *L. bocagei* | 100 | potamodromous | wide | 414.22 | temporary | south | connected | 0.000 | 0.00000 | 0.000 |
| Sado-Campilhas | *L. bocagei* | 100 | potamodromous | wide | 716.75 | temporary | south | connected | 0.395 | 0.00165 | 1.184 |
| Sado-Corona | *L. bocagei* | 100 | potamodromous | wide | 221.36 | temporary | south | connected | 0.000 | 0.00000 | 0.000 |
| Âncora | *A. oligolepis* | 16 | non-migratory | wide | 75.86 | permanent | north | unconnected | 1 | 0.00000 | 0 |
| Cabanas | *A. oligolepis* | 16 | non-migratory | wide | 12.40 | permanent | north | unconnected | 0.2684 | 0.00112 | 0.805 |
| Pego | *A. oligolepis* | 16 | non-migratory | wide | 10.36 | permanent | north | unconnected | 0.5809 | 0.00231 | 1.662 |
| Lima | *A. oligolepis* | 16 | non-migratory | wide | 1166.74 | permanent | north | unconnected | 0 | 0.00000 | 0 |
| Neiva | *A. oligolepis* | 16 | non-migratory | wide | 260.43 | permanent | north | unconnected | 0 | 0.00000 | 0 |
| Cávado | *A. oligolepis* | 16 | non-migratory | wide | 1609.97 | permanent | north | unconnected | 0.17 | 0.00060 | 0.435 |
| Ave | *A. oligolepis* | 16 | non-migratory | wide | 1390.34 | permanent | north | unconnected | 0.4853 | 0.00104 | 0.75 |
| Douro-Corgo | *A. oligolepis* | 16 | non-migratory | wide | 469.39 | permanent | north | connected | 0 | 0.00000 | 0 |
| Douro-Tua | *A. oligolepis* | 16 | non-migratory | wide | 3122.20 | permanent | north | connected | 0 | 0.00000 | 0 |
| Douro-Sabor | *A. oligolepis* | 16 | non-migratory | wide | 2377.22 | permanent | north | connected | 0.8478 | 0.00437 | 3.149 |
| Douro-Paiva | *A. oligolepis* | 16 | non-migratory | wide | 790.37 | permanent | north | connected | 0.0833 | 0.00023 | 0.167 |
| Douro-Coa | *A. oligolepis* | 16 | non-migratory | wide | 2520.69 | permanent | north | connected | 0 | 0.00000 | 0 |
| Vouga-Caima | *A. oligolepis* | 16 | non-migratory | wide | 211.06 | permanent | north | connected | 0.7662 | 0.00189 | 1.359 |
| Vouga-Sul | *A. oligolepis* | 16 | non-migratory | wide | 110.45 | permanent | north | connected | 0.2279 | 0.00049 | 0.353 |
| Vouga-Mel | *A. oligolepis* | 16 | non-migratory | wide | 94.81 | permanent | north | connected | 0 | 0.00000 | 0 |
| Vouga-Águeda | *A. oligolepis* | 16 | non-migratory | wide | 385.19 | permanent | north | connected | 0.2924 | 0.00096 | 0.69 |
| Mondego-Mortágua | *A. oligolepis* | 16 | non-migratory | wide | 198.97 | permanent | north | connected | 0.4158 | 0.00108 | 0.774 |
| Mondego-Dão | *A. oligolepis* | 16 | non-migratory | wide | 1127.77 | permanent | north | connected | 0.2807 | 0.00039 | 0.281 |
| Mondego-Arunca | *A. oligolepis* | 16 | non-migratory | wide | 729.47 | permanent | north | connected | 0.7059 | 0.00123 | 0.882 |
| Mondego-Corvo | *A. oligolepis* | 16 | non-migratory | wide | 228.97 | permanent | north | connected | 0.6397 | 0.00116 | 0.838 |
| Mondego-Ceira | *A. oligolepis* | 16 | non-migratory | wide | 503.19 | permanent | north | connected | 0.8088 | 0.00247 | 1.779 |
| Mondego-Alva | *A. oligolepis* | 16 | non-migratory | wide | 707.63 | permanent | north | connected | 0.8316 | 0.00180 | 1.295 |
| São Pedro | *A. oligolepis* | 16 | non-migratory | wide | 50.46 | temporary | south | unconnected | 0.4583 | 0.00064 | 0.458 |
| Alcoa | *A. oligolepis* | 16 | non-migratory | wide | 421.19 | temporary | south | unconnected | 0.205 | 0.00029 | 0.211 |
| Real | *A. oligolepis* | 16 | non-migratory | wide | 403.77 | temporary | south | unconnected | 0.521 | 0.00135 | 0.968 |
| Tejo-Zêzere Nabão | *A. oligolepis* | 16 | non-migratory | wide | 1016.84 | permanent | south | connected | 0.781 | 0.00372 | 2.676 |
| Lima | *P. duriense* | 34 | potamodromous | wide | 1166.74 | permanent | north | unconnected | 0.456 | 0.00190 | 1.368 |
| Neiva | *P. duriense* | 34 | potamodromous | wide | 260.43 | permanent | north | unconnected | 0.000 | 0.00000 | 0.000 |
| Cávado | *P. duriense* | 34 | potamodromous | wide | 1609.97 | permanent | north | unconnected | 0.467 | 0.00090 | 0.648 |
| Ave | *P. duriense* | 34 | potamodromous | wide | 1390.34 | permanent | north | unconnected | 0.529 | 0.00106 | 0.765 |
| Douro-Sousa | *P. duriense* | 34 | potamodromous | wide | 557.22 | permanent | north | connected | 0.468 | 0.00116 | 0.837 |
| Douro-Tâmega | *P. duriense* | 34 | potamodromous | wide | 2643.24 | permanent | north | connected | 0.838 | 0.00217 | 1.559 |
| Douro-Corgo | *P. duriense* | 34 | potamodromous | wide | 469.39 | permanent | north | connected | 0.847 | 0.00412 | 2.968 |
| Douro-Tua | *P. duriense* | 34 | potamodromous | wide | 3122.20 | permanent | north | connected | 0.836 | 0.00302 | 2.175 |
| Douro-Sabor | *P. duriense* | 34 | potamodromous | wide | 2377.22 | permanent | north | connected | 0.884 | 0.00302 | 2.174 |
| Douro-Paiva | *P. duriense* | 34 | potamodromous | wide | 790.37 | permanent | north | connected | 0.000 | 0.00000 | 0.000 |
| Douro-Távora | *P. duriense* | 34 | potamodromous | wide | 531.87 | permanent | north | connected | 0.581 | 0.00178 | 1.279 |
| Vouga-Caima | *P. duriense* | 34 | potamodromous | wide | 211.06 | permanent | north | connected | 0.325 | 0.00135 | 0.975 |
| Vouga-Sul | *P. duriense* | 34 | potamodromous | wide | 110.45 | permanent | north | connected | 0.529 | 0.00221 | 1.588 |
| Vouga-Águeda | *P. duriense* | 34 | potamodromous | wide | 385.19 | permanent | north | connected | 0.118 | 0.00033 | 0.235 |
| Mondego-Mortágua | *P. polylepis* | 33 | potamodromous | wide | 198.97 | permanent | north | connected | 0.467 | 0.00065 | 0.467 |
| Mondego-Dão | *P. polylepis* | 33 | potamodromous | wide | 1127.77 | permanent | north | connected | 0.000 | 0.00000 | 0.000 |
| Mondego-Arunca | *P. polylepis* | 33 | potamodromous | wide | 729.47 | permanent | north | connected | 0.000 | 0.00000 | 0.000 |
| Mondego-Corvo | *P. polylepis* | 33 | potamodromous | wide | 228.97 | permanent | north | connected | 0.000 | 0.00000 | 0.000 |
| Mondego-Alva | *P. polylepis* | 33 | potamodromous | wide | 707.63 | permanent | north | connected | 0.111 | 0.00015 | 0.111 |
| Tejo-Erges | *P. polylepis* | 33 | potamodromous | wide | 592.83 | permanent | south | connected | 0.628 | 0.00132 | 0.948 |
| Tejo-Ponsul | *P. polylepis* | 33 | potamodromous | wide | 1456.70 | permanent | south | connected | 0.676 | 0.00230 | 1.657 |
| Tejo-Ocreza | *P. polylepis* | 33 | potamodromous | wide | 1429.42 | permanent | south | connected | 0.595 | 0.00095 | 0.686 |
| Tejo-Zêzere Nabão | *P. polylepis* | 33 | potamodromous | wide | 1016.84 | permanent | south | connected | 0.737 | 0.00193 | 1.389 |
| Tejo-Almonda | *P. polylepis* | 33 | potamodromous | wide | 237.16 | permanent | south | connected | 0.684 | 0.00121 | 0.868 |
| Tejo-Alviela | *P. polylepis* | 33 | potamodromous | wide | 371.78 | permanent | south | connected | 0.309 | 0.00086 | 0.618 |
| Tejo-Sever | *P. polylepis* | 33 | potamodromous | wide | 300.04 | temporary | south | connected | 0.542 | 0.00081 | 0.583 |
| Tejo-Nisa | *P. polylepis* | 33 | potamodromous | wide | 264.16 | temporary | south | connected | 0.569 | 0.00099 | 0.712 |
| Tejo-Muge | *P. polylepis* | 33 | potamodromous | wide | 703.14 | temporary | south | connected | 0.634 | 0.00343 | 2.471 |
| Tejo-Sorraia | *P. polylepis* | 33 | potamodromous | wide | 7616.87 | temporary | south | connected | 0.862 | 0.00208 | 1.495 |
| Sado-Campilhas | *P. polylepis* | 33 | potamodromous | wide | 716.75 | temporary | south | connected | 0.125 | 0.00017 | 0.125 |
| Guadiana-Chança | *P. willkommii* | 32 | potamodromous | restricted | 485.05 | temporary | south | connected | 0.863 | 0.00314 | 2.263 |
| Guadiana-Caia | *P. willkommii* | 32 | potamodromous | restricted | 794.57 | temporary | south | connected | 0.739 | 0.00143 | 1.033 |
| Guadiana-Degebe | *P. willkommii* | 32 | potamodromous | restricted | 1538.40 | temporary | south | connected | 0.784 | 0.00219 | 1.575 |
| Guadiana-Cobres | *P. willkommii* | 32 | potamodromous | restricted | 1156.23 | temporary | south | connected | 0.760 | 0.00249 | 1.789 |
| Guadiana-Oeiras | *P. willkommii* | 32 | potamodromous | restricted | 498.60 | temporary | south | connected | 0.766 | 0.00177 | 1.275 |
| Guadiana-Vascão | *P. willkommii* | 32 | potamodromous | restricted | 443.32 | temporary | south | connected | 0.807 | 0.00309 | 2.222 |
| Minho | *S. carolitertii* | 23 | non-migratory | wide | 804.47 | permanent | north | unconnected | 0 | 0.00000 | 0 |
| Pego | *S. carolitertii* | 23 | non-migratory | wide | 10.36 | permanent | north | unconnected | 0 | 0.00000 | 0 |
| Lima | *S. carolitertii* | 23 | non-migratory | wide | 1166.74 | permanent | north | unconnected | 0.4143 | 0.00062 | 0.448 |
| Neiva | *S. carolitertii* | 23 | non-migratory | wide | 260.43 | permanent | north | unconnected | 0.3957 | 0.00055 | 0.396 |
| Cávado | *S. carolitertii* | 23 | non-migratory | wide | 1609.97 | permanent | north | unconnected | 0.5543 | 0.00084 | 0.601 |
| Ave | *S. carolitertii* | 23 | non-migratory | wide | 1390.34 | permanent | north | unconnected | 0.1 | 0.00014 | 0.1 |
| Douro-Sousa | *S. carolitertii* | 23 | non-migratory | wide | 557.22 | permanent | north | connected | 0.4421 | 0.00061 | 0.442 |
| Douro-Tâmega | *S. carolitertii* | 23 | non-migratory | wide | 2643.24 | permanent | north | connected | 0.6644 | 0.00153 | 1.101 |
| Douro-Sabor | *S. carolitertii* | 23 | non-migratory | wide | 2377.22 | permanent | north | connected | 0.625 | 0.00108 | 0.779 |
| Douro-Paiva | *S. carolitertii* | 23 | non-migratory | wide | 790.37 | permanent | north | connected | 0 | 0.00000 | 0 |
| Douro-Távora | *S. carolitertii* | 23 | non-migratory | wide | 531.87 | permanent | north | connected | 0.625 | 0.00191 | 1.375 |
| Douro-Coa | *S. carolitertii* | 23 | non-migratory | wide | 2520.69 | permanent | north | connected | 0 | 0.00000 | 0 |
| Vouga-Caima | *S. carolitertii* | 23 | non-migratory | wide | 211.06 | permanent | north | connected | 0 | 0.00000 | 0 |
| Vouga-Sul | *S. carolitertii* | 23 | non-migratory | wide | 110.45 | permanent | north | connected | 0.0588 | 0.00008 | 0.059 |
| Vouga-Águeda | *S. carolitertii* | 23 | non-migratory | wide | 385.19 | permanent | north | connected | 0.6901 | 0.00119 | 0.854 |
| Mondego-Mortágua | *S. carolitertii* | 23 | non-migratory | wide | 198.97 | permanent | north | connected | 0.6476 | 0.00106 | 0.762 |
| Mondego-Dão | *S. carolitertii* | 23 | non-migratory | wide | 1127.77 | permanent | north | connected | 0.2047 | 0.00072 | 0.515 |
| Mondego-Corvo | *S. carolitertii* | 23 | non-migratory | wide | 228.97 | permanent | north | connected | 0.4248 | 0.00059 | 0.425 |
| Mondego-Ceira | *S. carolitertii* | 23 | non-migratory | wide | 503.19 | permanent | north | connected | 0.7524 | 0.01897 | 2.952 |
| Mondego-Alva | *S. carolitertii* | 23 | non-migratory | wide | 707.63 | permanent | north | connected | 0.4561 | 0.00158 | 1.135 |
| Alcoa | *S. carolitertii* | 23 | non-migratory | wide | 421.19 | temporary | south | unconnected | 0.1 | 0.00014 | 0.1 |
| Lizandro | *S. pyrenaicus* | 18 | non-migratory | wide | 167.58 | temporary | south | unconnected | 0.6421 | 0.00219 | 1.574 |
| Samarra | *S. pyrenaicus* | 18 | non-migratory | wide | 19.78 | temporary | south | unconnected | 0.1857 | 0.00250 | 1.8 |
| Colares | *S. pyrenaicus* | 18 | non-migratory | wide | 49.65 | temporary | south | unconnected | 0.4524 | 0.00066 | 0.476 |
| Jamor | *S. pyrenaicus* | 18 | non-migratory | wide | 44.22 | temporary | south | unconnected | 0.1 | 0.00083 | 0.6 |
| Tejo-Erges | *S. pyrenaicus* | 18 | non-migratory | wide | 592.83 | permanent | south | connected | 0.6993 | 0.00445 | 3.275 |
| Tejo-Ponsul | *S. pyrenaicus* | 18 | non-migratory | wide | 1456.70 | permanent | south | connected | 0.8309 | 0.00431 | 3.103 |
| Tejo-Almonda | *S. pyrenaicus* | 18 | non-migratory | wide | 237.16 | permanent | south | connected | 0.8381 | 0.00228 | 1.642 |
| Tejo-Ota | *S. pyrenaicus* | 18 | non-migratory | wide | 287.42 | permanent | south | connected | 0.6368 | 0.00170 | 1.226 |
| Tejo-Grande da pipa | *S. pyrenaicus* | 18 | non-migratory | wide | 118.38 | permanent | south | connected | 0.7105 | 0.00612 | 4.405 |
| Tejo-Muge | *S. pyrenaicus* | 18 | non-migratory | wide | 703.14 | temporary | south | connected | 0.6952 | 0.00291 | 2.095 |
| Sado-Odivelas | *S. pyrenaicus* | 18 | non-migratory | wide | 737.21 | temporary | south | connected | 0.1 | 0.00236 | 1.7 |
| Sado-S.Martinho | *S. pyrenaicus* | 18 | non-migratory | wide | 246.39 | temporary | south | connected | 0.2789 | 0.00623 | 4.484 |
| Sado-Campilhas | *S. pyrenaicus* | 18 | non-migratory | wide | 716.75 | temporary | south | connected | 0.3684 | 0.00054 | 0.386 |
| Gilão | *S. pyrenaicus* | 18 | non-migratory | wide | 223.48 | temporary | south | unconnected | 0 | 0.00000 | 1.038 |
| Guadiana-Caia | *S. pyrenaicus* | 18 | non-migratory | wide | 794.57 | temporary | south | connected | 0.8363 | 0.00197 | 0.4 |
| Guadiana-Cobres | *S. pyrenaicus* | 18 | non-migratory | wide | 1156.23 | temporary | south | connected | 0.5789 | 0.00102 | 0 |
| Guadiana-Oeiras | *S. pyrenaicus* | 18 | non-migratory | wide | 498.60 | temporary | south | connected | 0.8182 | 0.00197 | 0 |
| Guadiana-Odeleite | *S. pyrenaicus* | 18 | non-migratory | wide | 411.53 | temporary | south | connected | 0.8889 | 0.00398 | 2.326 |
| Mira | *S. torgalensis* | 16 | non-migratory | restricted | 1575.11 | temporary | south | unconnected | 0.2714 | 0.00144 | 0 |
| Seixe | *S. aradensis* | 14 | non-migratory | restricted | 255.44 | temporary | south | unconnected | 0.3684 | 0.00056 | 0 |
| Aljezur | *S. aradensis* | 14 | non-migratory | restricted | 181.08 | temporary | south | unconnected | 0 | 0.00000 | 1.415 |
| Alvor | *S. aradensis* | 14 | non-migratory | restricted | 256.83 | temporary | south | unconnected | 0 | 0.00000 | 0.737 |
| Arade | *S. aradensis* | 14 | non-migratory | restricted | 975.59 | temporary | south | unconnected | 0.7895 | 0.00323 | 1.415 |
| Quarteira | *S. aradensis* | 14 | non-migratory | restricted | 405.60 | temporary | south | unconnected | 0 | 0.00000 | 2.863 |
| Tejo-Ocreza | *L. comizo* | 100 | potamodromous | wide | 1429.42 | permanent | south | connected | 0 | 0.00000 | 0 |
| Guadiana-Ardila | *L. comizo* | 100 | potamodromous | wide | 884.72 | temporary | south | connected | 0.1109 | 0.00015 | 0.111 |
| Guadiana-Chança | *L. comizo* | 100 | potamodromous | wide | 485.05 | temporary | south | connected | 0 | 0.00000 | 0 |
| Guadiana-Degebe | *L. comizo* | 100 | potamodromous | wide | 794.57 | temporary | south | connected | 0.125 | 0.00017 | 0.125 |
| Guadiana-Cobres | *L. comizo* | 100 | potamodromous | wide | 1538.40 | temporary | south | connected | 0.1111 | 0.00015 | 0.111 |
| Guadiana-Vascão | *L. comizo* | 100 | potamodromous | wide | 443.32 | temporary | south | connected | 0 | 0.00000 | 0 |
| Mira | *L. sclateri* | 46 | potamodromous | wide | 1575.11 | temporary | south | unconnected | 0.4583 | 0.00064 | 0.458 |
| Seixe | *L. sclateri* | 46 | potamodromous | wide | 255.44 | temporary | south | unconnected | 0.000 | 0.00000 | 0.000 |
| Arade | *L. sclateri* | 46 | potamodromous | wide | 181.08 | temporary | south | unconnected | 0.000 | 0.00000 | 0.000 |
| Gilão | *L. sclateri* | 46 | potamodromous | wide | 223.48 | temporary | south | unconnected | 0.419 | 0.00058 | 0.419 |
| Guadiana-Ardila | *L. sclateri* | 46 | potamodromous | wide | 884.72 | temporary | south | connected | 0.3305 | 0.00187 | 1.345 |
| Guadiana-Chança | *L. sclateri* | 46 | potamodromous | wide | 485.05 | temporary | south | connected | 0.2652 | 0.00037 | 0.265 |
| Guadiana-Oeiras | *L. sclateri* | 46 | potamodromous | wide | 498.60 | temporary | south | connected | 0.3117 | 0.00043 | 0.312 |
| Guadiana-Vascão | *L. sclateri* | 46 | potamodromous | wide | 443.32 | temporary | south | connected | 0.2984 | 0.00043 | 0.311 |
| Guadiana-Odeleite | *L. sclateri* | 46 | potamodromous | wide | 411.53 | temporary | south | connected | 0.5367 | 0.00439 | 3.16 |
| Lizandro | *I. lusitanicum* | 13 | non-migratory | wide | 167.58 | temporary | south | unconnected | 0 | 0.00000 | 0 |
| Samarra | *I. lusitanicum* | 13 | non-migratory | wide | 19.78 | temporary | south | unconnected | 0 | 0.00000 | 0 |
| Colares | *I. lusitanicum* | 13 | non-migratory | wide | 49.65 | temporary | south | unconnected | 0.1111 | 0.00031 | 0.222 |
| Barcarena | *I. lusitanicum* | 13 | non-migratory | wide | 34.22 | temporary | south | unconnected | 0 | 0.00000 | 0 |
| Jamor | *I. lusitanicum* | 13 | non-migratory | wide | 44.22 | temporary | south | unconnected | 0 | 0.00000 | 0 |
| Tejo-Alviela | *I. lusitanicum* | 13 | non-migratory | wide | 371.78 | permanent | south | connected | 0.5515 | 0.00086 | 0.618 |
| Tejo-Maior | *I. lusitanicum* | 13 | non-migratory | wide | 957.05 | permanent | south | connected | 0.822 | 0.00216 | 1.552 |
| Tejo-Ota | *I. lusitanicum* | 13 | non-migratory | wide | 287.42 | permanent | south | connected | 0.5556 | 0.00166 | 1.193 |
| Tejo-Grande da pipa | *I. lusitanicum* | 13 | non-migratory | wide | 118.38 | permanent | south | connected | 0.5947 | 0.00171 | 1.232 |
| Tejo-Trancão | *I. lusitanicum* | 13 | non-migratory | wide | 279.02 | permanent | south | connected | 0.5146 | 0.00143 | 1.029 |
| Tejo-Coina | *I. lusitanicum* | 13 | non-migratory | wide | 82.14 | temporary | south | connected | 0.5 | 0.00200 | 1.437 |
| Sado-Odivelas | *I. lusitanicum* | 13 | non-migratory | wide | 737.21 | temporary | south | connected | 0.6842 | 0.00146 | 1.053 |
| Sado-S.Martinho | *I. lusitanicum* | 13 | non-migratory | wide | 246.39 | temporary | south | connected | 0.2941 | 0.00490 | 3.529 |
| Sado-Grandola | *I. lusitanicum* | 13 | non-migratory | wide | 262.59 | temporary | south | connected | 0.7947 | 0.00183 | 1.316 |
| Mira | *I. almacai* | 15 | non-migratory | restricted | 1575.11 | temporary | south | unconnected | 0.4421 | 0.00061 | 0.442 |
| Arade | *I. almacai* | 15 | non-migratory | restricted | 975.59 | temporary | south | unconnected | 0.5105 | 0.00075 | 0.542 |
| Guadiana-Chança | *I. lemmingii* | 15 | non-migratory | restricted | 485.05 | temporary | south | connected | 0.5752 | 0.00096 | 0.693 |
| Guadiana-Degebe | *I. lemmingii* | 15 | non-migratory | restricted | 1538.40 | temporary | south | connected | 0.5088 | 0.00200 | 1.439 |
| Guadiana-Odeleite | *I. lemmingii* | 15 | non-migratory | restricted | 411.53 | temporary | south | connected | 0.7263 | 0.00222 | 0.002 |
| Alcabrichel | *A. occidentale* | 10 | non-migratory | restricted | 150.78 | temporary | south | unconnected | 0.8142 | 0.00198 | 1.423 |
| Sizandro | *A. occidentale* | 10 | non-migratory | restricted | 334.35 | temporary | south | unconnected | 0.0000 | 0.00000 | 0 |
| Safarujo | *A. occidentale* | 10 | non-migratory | restricted | 59.21 | temporary | south | unconnected | 0.1176 | 0.00038 | 0.273 |
| Guadiana-Ardila | *A. hispanica* | 8 | non-migratory | restricted | 884.72 | temporary | south | connected | 0.693 | 0.00536 | 3.861 |
| Guadiana-Chança | *A. hispanica* | 8 | non-migratory | restricted | 485.05 | temporary | south | connected | 0.629 | 0.00078 | 0.562 |
| Guadiana-Vascão | *A. hispanica* | 8 | non-migratory | restricted | 443.32 | temporary | south | connected | 0.548 | 0.00102 | 0.735 |
| Guadiana-Odeleite | *A. hispanica* | 8 | non-migratory | restricted | 411.53 | temporary | south | connected | 0.569 | 0.00252 | 1.811 |
| Guadiana-Foupana | *A. hispanica* | 8 | non-migratory | restricted | 359.56 | temporary | south | connected | 0.634 | 0.00249 | 1.794 |
|  |  |  |  |  |  |  |  |  |  |  |  |
|  |  |  |  |  |  |  |  |  |  |  |  |
|  |  |  |  |  |  |  |  |  |  |  |  |
|  |  |  |  |  |  |  |  |  |  |  |  |
|  |  |  |  |  |  |  |  |  |  |  |  |
